# Depletion of central memory CD8^+^ T cells might impede the antitumor therapeutic effect of Mogamulizumab

**DOI:** 10.1038/s41467-021-27574-0

**Published:** 2021-12-14

**Authors:** Yuka Maeda, Hisashi Wada, Daisuke Sugiyama, Takuro Saito, Takuma Irie, Kota Itahashi, Kodai Minoura, Susumu Suzuki, Takashi Kojima, Kazuhiro Kakimi, Jun Nakajima, Takeru Funakoshi, Shinsuke Iida, Mikio Oka, Teppei Shimamura, Toshihiko Doi, Yuichiro Doki, Eiichi Nakayama, Ryuzo Ueda, Hiroyoshi Nishikawa

**Affiliations:** 1 Division of Cancer Immunology, Research Institute/Exploratory Oncology Research & Clinical Trial Center (EPOC), National Cancer Center, Tokyo, 104-0045/Chiba, 277-8577 Japan; 2 Department of Clinical Research in Tumor Immunology, Osaka University Graduate School of Medicine, Osaka, 565-0871 Japan; 3 Department of Immunology, Nagoya University Graduate School of Medicine, Nagoya, 466–8550 Japan; 4 Department of Gastroenterological Surgery, Osaka University Graduate School of Medicine, Osaka, 565-0871 Japan; 5 Department of Systems Biology, Nagoya University Graduate School of Medicine, Nagoya, 466–8550 Japan; 6 Department of Tumor Immunology, Aichi Medical University, Aichi, 480-1195 Japan; 7 Department of Gastrointestinal Oncology, National Cancer Center Hospital East, Chiba, 277-8577 Japan; 8 Department of Immunotherapeutics, The University of Tokyo Hospital, Tokyo, 113-8655 Japan; 9 Department of Thoracic Surgery, Graduate School of Medicine, The University of Tokyo, Tokyo, 113-8655 Japan; 10 Department of Dermatology, Keio University School of Medicine, Tokyo, 160-8582 Japan; 11 Department of Hematology and Oncology, Nagoya City University Institute of Medical and Pharmaceutical Sciences, Nagoya, 467-8601 Japan; 12 Department of Respiratory Medicine, Kawasaki Medical School, Okayama 701-0192, Japan; 13 Faculty of Health and Welfare, Kawasaki University of Medical Welfare, Okayama, 701-0192 Japan

**Keywords:** Immunosuppression, Cancer immunotherapy, Molecular medicine, Immunoediting

## Abstract

Regulatory T (Treg) cells are important negative regulators of immune homeostasis, but in cancers they tone down the anti-tumor immune response. They are distinguished by high expression levels of the chemokine receptor CCR4, hence their targeting by the anti-CCR4 monoclonal antibody mogamulizumab holds therapeutic promise. Here we show that despite a significant reduction in peripheral effector Treg cells, clinical responses are minimal in a cohort of patients with advanced CCR4-negative solid cancer in a phase Ib study (NCT01929486). Comprehensive immune-monitoring reveals that the abundance of CCR4-expressing central memory CD8^+^ T cells that are known to play roles in the antitumor immune response is reduced. In long survivors, characterised by lower CCR4 expression in their central memory CD8^+^ T cells possessed and/or NK cells with an exhausted phenotype, cell numbers are eventually maintained. Our study thus shows that mogamulizumab doses that are currently administered to patients in clinical studies may not differentiate between targeting effector Treg cells and central memory CD8^+^ T cells, and dosage refinement might be necessary to avoid depletion of effector components during immune therapy.

## Abstract (web summary)

Elimination of regulatory T cells via the anti-CCR4 monoclonal antibody, mogamulizumab, is expected to augment anti-tumour immune response. Authors show here that although regulatory T cell targeting is successful, clinical improvement remains minimal in patients with solid tumours due to concomitant and unintended depletion of central memory CD8^+^ T cells.

## Introduction

Genetic instability is an evolving hallmark of cancer cells; consequently, cancer cells frequently possess gene alterations that generate abnormal proteins^1^. The immune system recognizes these abnormal proteins as non-self antigens (termed neoantigens) and elicits antitumor immune responses (cancer immunosurveillance)^2,3^. Therefore, clinically apparent cancer compels two crucial processes during the development, namely, (1) the reduction of immunogenicity by decreasing the expression of abnormal proteins that can readily induce immune responses, and (2) the establishment of immune escape mechanisms involved in multiple immune suppressive machineries such as immunosuppressive cells and immunosuppressive molecules^4^. Cancer immunotherapy represented by immune checkpoint blockade (ICB) unleashes the effects of effector T cells and kills cancer cells, resulting in tumor regression across multiple cancer types^5^. However, more than half of patients treated with cancer immunotherapy fail to respond to ICB, even after combination therapies; therefore, developing more effective therapies is urgently required^6^.

Regulatory T (Treg) cells expressing the transcription factor forkhead box P3 (FoxP3) are an immunosuppressive CD4^+^ T cell subset that are indispensable for the maintenance of self-tolerance and immune homeostasis^7–9^. In addition to immune checkpoint molecules, Treg cells hinder protective cancer immunosurveillance in healthy individuals and hamper effective antitumor immune responses in tumor-bearing hosts. Thus, a high frequency of Treg cells and their dominance to effector T cells in the tumor microenvironment (TME) are associated with poor prognosis in various types of cancer^10–13^. Hence, Treg cells are an attractive therapeutic target for cancer immunotherapy. In animal models, selective depletion of Treg cells has been reported to robustly augment antitumor immune responses and contribute to tumor eradication^13–15^. In humans, previous studies have examined the effects of Treg cell depletion by targeting CD25 with antibodies or a recombinant protein composed of IL-2 and the active domain of the diphtheria toxin, as Treg cells were originally identified as CD25^+^CD4^+^ T cells^7^. However, anti-CD25 mAb depletes both effector T cells and Treg cells; consequently, neither antitumor T cell responses nor antibody production is observed^13–17^. As CD25 expression is induced upon the activation of effector T cells, CD25-targeted Treg cell depletion may be accompanied by a reduction in effector T cells, implying the importance of more selective depletion methods^18^. Moreover, given the crucial role of Treg cells in self-tolerance, Treg cell depletion on the whole can trigger autoimmunity in animal models^8,15,19^. Therefore, an important issue is how Treg cells can be controlled to evoke and augment antitumor immunity without inducing deleterious autoimmunity, this strongly indicates the necessity of developing Treg cell depletion methods with superior selectivity to eliminate tumor-infiltrating Treg cells.

To identify the molecules that can specifically target tumor-infiltrating Treg cells, the accurate identification of Treg cells is essential. FoxP3^+^ T cells in humans are heterogeneous in phenotype and function due to the upregulation of FoxP3 in naive T cells upon T-cell receptor stimulation^9,13,20^. Accordingly, human FoxP3^+^CD4^+^ T cells are fractionated into the following three subsets based on the expression levels of the naive T cell marker CD45RA and FoxP3 or CD25: Fraction (Fr.) I naive Treg cells (CD45RA^+^CD25^low^FoxP3^low^CD4^+^); Fr. II effector Treg (eTreg) cells (CD45RA^−^CD25^high^FoxP3^high^CD4^+^); and Fr. III non-Treg cells (CD45RA^−^CD25^low^FoxP3^low^CD4^+^). Fr. II eTreg cells, which possess high CTLA-4 expression and strong immune suppressive activity, are the predominant tumor-infiltrating FoxP3^+^CD4^+^ T cells that are found in the majority of cancers^9,13,20^. We have previously reported that C-C chemokine receptor (CCR) 4 is highly expressed in eTreg cells, probably through the involvement of C-C motif chemokine 22 (CCL22) for the infiltration of Treg cells into the TME, and that Treg cell depletion by targeting of CCR4 induces increased tumor antigen-specific CD4^+^ and CD8^+^ T cell responses^13,21^.

A phase 1a clinical trial of Treg cell depletion by the administration of anti-CCR4 mAb (mogamulizumab, KW-0761) for advanced or recurrent solid tumor patients revealed a significant reduction of eTreg cells in the peripheral blood^22^. While humoral responses against NY‐ESO‐1 and XAGE1 antigens were observed in patients with NY‐ESO‐1 and XAGE1-expressing tumors, respectively, clinical responses were unfortunately not observed in most patients^22^. In this study, we aimed at addressing comprehensive immunological changes pre- and post-mogamulizumab administration through analyzing patient samples enrolled in a phase 1b clinical trial^23^ with mogamulizumab monotherapy using multicolor flow cytometry and CyTOF. We found an unexpected reduction of central memory CD8^+^ T cells, which reportedly play an important role in antitumor activity in cancer immunotherapy^24,25^, harboring CCR4 expression accompanied by Treg cell reduction after mogamulizumab administration; this indicates the importance of developing more specific Treg cell-targeted therapies.

## Results

### Mogamulizumab administration is well-tolerated but induces limited clinical efficacy

Thirty-nine patients with advanced CCR4-negative solid cancer received mogamulizumab at two dosages (0.1 and 1.0 mg/kg) cohorts in this phase Ib study (Table 1)^23^. Their median age was 65 years, and 11 esophageal cancer, 12 lung cancer, six malignant melanoma, five gastric cancer and five ovarian cancer patients were administered mogamulizumab 2–23 times. In the 0.1 mg/kg cohort, a total of 198 adverse events (AEs) and 65 mogamulizumab-related AEs were observed, whereas in the 1.0 mg/kg cohort, a total of 126 AEs and 49 mogamulizumab-related AEs were noted. Among the mogamulizumab-related AEs, skin disorders and lymphopenia were the most frequently observed (Table 2).

**Table 1:** Patient characteristics and clinical responses.

		0.1 mg/kg	1.0 mg/kg	Total
		(*n* = 20)	(*n* = 19)	(*n* = 39)
Sex	Male/female	12/8	13/6	25/14
Age (year-old)	Median (range)	66 (47–85)	65 (45–80)	65 (45–85)
BMI (kg/m^2^)	Mean (sd)	20.6 (3.2)	20.5 (2.7)	20.5 (3.0)
Cancer organ				
	Esophagus	6	5	11
	Lung	6	6	12
	Skin	4	2	6
	Stomach	2	3	5
	Ovary	2	3	5
ECOG	PS; 0/1/2	9/10/1	10/8/1	19/18/2
Prior therapies				
	Chemotherapy	20	18	38
	Surgery	11	14	25
	Radiation	8	6	14
	Other therapy	3	5	8
Total # of dose mogamulizumab				
	Median (range)	8 (2–14)	8 (2–23)	8 (2–23)
Early escape				
	Drop-out	1	0	1
	Death	2	2	4
	Discontinuation	3	3	6
Clinical response				
	PR	0	1	1
	SD	3	2	5
	PD	16	15	31
PFS (days) (RECIST)	Median (range)	67 (21–96)	65 (25–491)	66 (21–491)
OS (days)	Median (range)	271 (21–498)	272 (25–511)	272 (21–511)

**Table 2:** Adverse effects of mogamulizumab administration.

	0.1 mg/kg (*n* = 20)		1.0 mg/kg (*n* = 19)		Total (*n* = 39)	
	Cases	Events	Cases	Events	Cases	Events
AEs	20	198	19	126	39	324
Related AE	19	65	17	49	36	114
Grade 1/2/3/4	16/12/6/1	34/23/7/1	10/14/4/2	23/20/4/2	26/26/10/3	57/43/11/3
*: Overlapping	*		*		*	
decreased lymphocytes		15		11		25
Grade 1/2/3/4		1/10/3/1		0/7/2/2		1/17/4/3
dermal disorders		15		11		26
Grade 1/2/3/4		9/5/1/0		5/6/0/0		14/11/1/0
Drug eruption, Grade 2				1		1
Asteatotic eczema, Grade 1		1				1
Erythema, Grade 1		1				1
Papule, Grade 2				1		1
Pruritus, Grade 1		1				1
Rash, Grade 1/2		1/4		2/3		3/7
Rash maculo-papular, Grade 1/2/3		5/1/1		3/1/0		8/2/1
other adverse effects						
increased alanine aminotransferase, Grade 1/3		3/0/1			3/0/1	
decreased appetite, Grade 1				1		1
increased aspartate aminotransferase, Grade 1/3		2/0/1			2/0/1	
Hypophosphatasemia, Grade 3				1		0/0/3
increased gamma-glutamyl transferase, Grade 3		1				0/0/3
hypophosphatasemia, Grade 1		1				1

One PR esophageal cancer patient and five SD lung or esophageal cancer patients were confirmed as exhibiting objective clinical responses according to the RECIST criteria. The median PFS and OS were 67 and 271 days in the 0.1 mg/kg cohort and 65 and 272 days in the 1.0 mg/kg cohort, respectively (Table 1 and Supplementary Fig. 1)^23^.

### CyTOF analyses reveal the comprehensive immunological features after mogamulizumab treatment

**Fig. 1:**
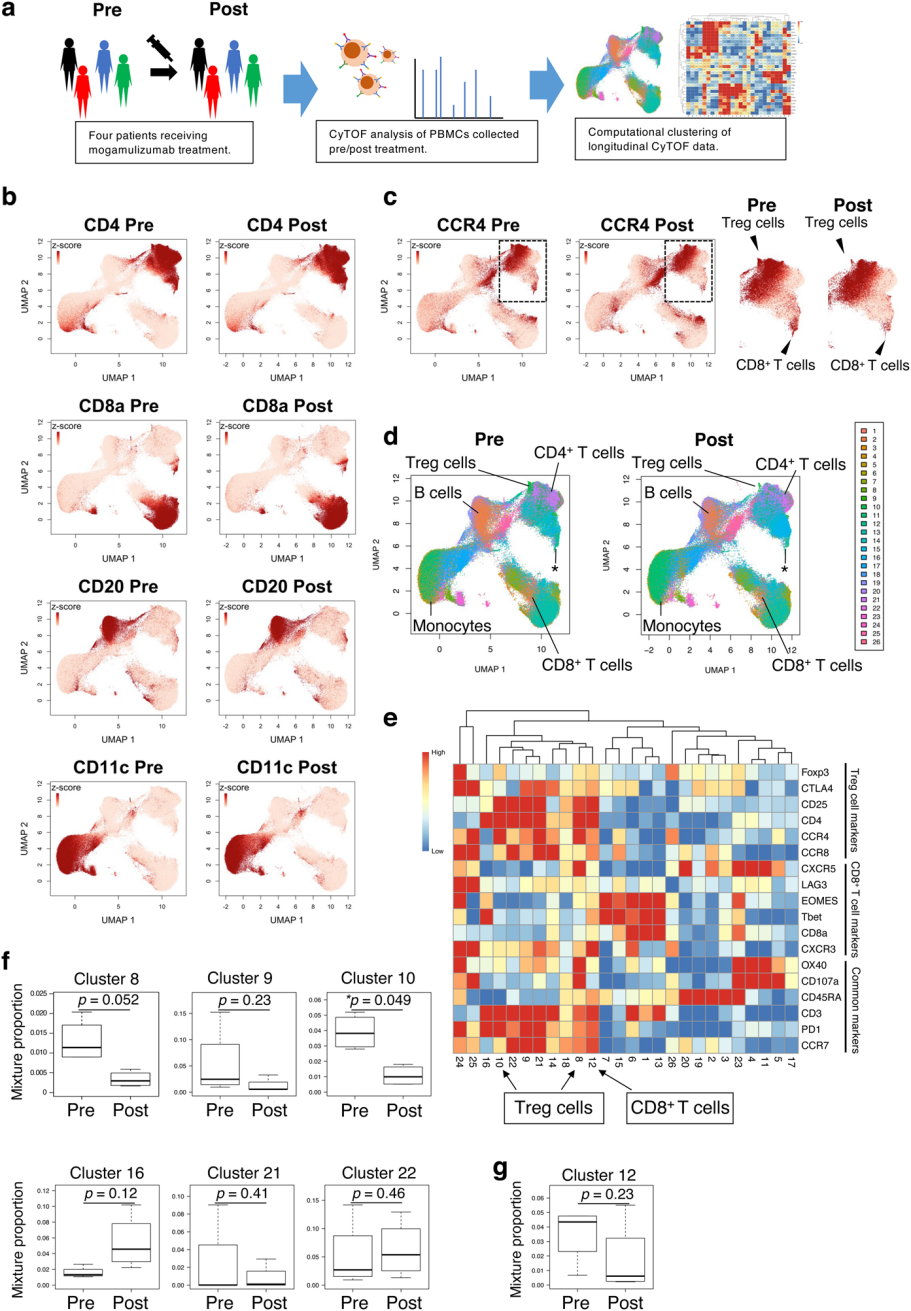
A comprehensive immunological landscape is uncovered by longitudinal CyTOF data obtained from patients treated with mogamulizumab. **a** Schematic overview of the CyTOF analyses. PBMCs (*n* = 4) obtained from pre- and post-mogamulizumab treatment were subjected to CyTOF. **b** UMAP projection of cells from pre- and post-mogamulizumab treatment samples is colored according to their scaled expression levels of markers for different cell populations: CD4^+^ T cells, CD8^+^ T cells, FoxP3^+^ T cells, CD20 for B cells, CD11c for monocytes. **c** The UMAP projection is colored according to the scaled expression levels of CCR4. The panels on the right represent the enlarged CD4^+^ cell populations to clearly show the changes in the expression levels of CCR4 pre- and post-mogamulizumab treatment samples. Black arrowheads represent Treg cells and CD8^+^ T cells. Each dot represents a single cell. Colors were saturated at z-cores 1 and 0 for visualization. **d** The UMAP projection is colored according to the cluster assignment by CYBERTRACK2.0. **e**. Heatmap generated by CYBERTRACK2.0. The rows and columns represent markers and clusters, respectively. Black arrowheads represent Treg cells and CD8^+^ T cells as in (**c**). **f** Boxplots representing the proportions of CD4^+^ clusters (clusters 8, *p* = 0.052; 9, *p* = 0.23; 10, *p = 0.049; 16, *p* = 0.12; 21, *p* = 0.41 and 22, *p* = 0.46) at pre- and post-mogamulizumab treatment (*n* = 4). **g** Boxplot representing the proportions of cluster 12 (*p* = 0.23), which contains CD8^+^ T cell populations at pre- and post-mogamulizumab treatment (n = 4). The center line indicates the median, and the box limits indicate the 1st and 3rd quartiles. Whiskers extend to the 1.5x interquartile range. In (**f**) and (**g**), two-sided paired Student’s t-test was used. *p < 0.05. Source data are provided as a Source Data file.

The unexpected observation of limited clinical responses prompted us to investigate the comprehensive immunological features after mogamulizumab treatment^22^. We subjected pre- (within two weeks before treatment) and post-treatment peripheral blood mononuclear cell (PBMC) samples from four patients (one PR and three PD) (Supplementary table 1), from whom sufficient amounts of samples were available to CyTOF analyses (Fig. 1a). Comprehensive immunological analyses with CyTOF showed a decrease in CCR4-positive cells after mogamulizumab administration irrespective of the mogamulizumab dosage (0.1 and 1.0 mg/kg). In particular, mogamulizumab significantly reduced some subpopulations of CD4^+^ T cells such as Treg cells, although most CD4^+^ T cell populations were increased, which was in line with the findings of our previous reports (Fig. 1b, c)^22^. Unexpectedly, while most CD8^+^ T cell populations were increased, a subpopulation of CD8^+^ T cells was decreased after mogamulizumab treatment (Fig. 1c). Unbiased clustering with CYBERTRACK further revealed a reduction in several finely clustered cell populations, such as cluster 8 (*p* = 0.052) and cluster 10 (*p* = 0.049) (containing Treg cells). In addition, a decrease and an increase, although not significant, were observed in cluster 12 (*p* = 0.23) (containing CD8^+^ T cells) and cluster 16 (*p* = 0.16) (containing activated non-Treg CD4^+^ T cells expressing PD-1), respectively (Fig. 1d–g and Supplementary Fig. 2a–c). The cluster 12 among CD8^+^ T cell clusters (clusters 1, 6, 12, 13 and 14) was only decreased after mogamulizumab administration (Fig. 1f, g). The frequency of other immune cells, such as B cells and monocytes, was not significantly changed after mogamulizumab administration (Fig. 1b, d, e and Supplementary Fig. 2a–c). These data suggest that mogamulizumab effectively depletes Treg cells as expected, but a subpopulation of CD8^+^ T cells may also be influenced.

### Mogamulizumab treatment efficiently depletes Treg cells

**Fig. 2:**
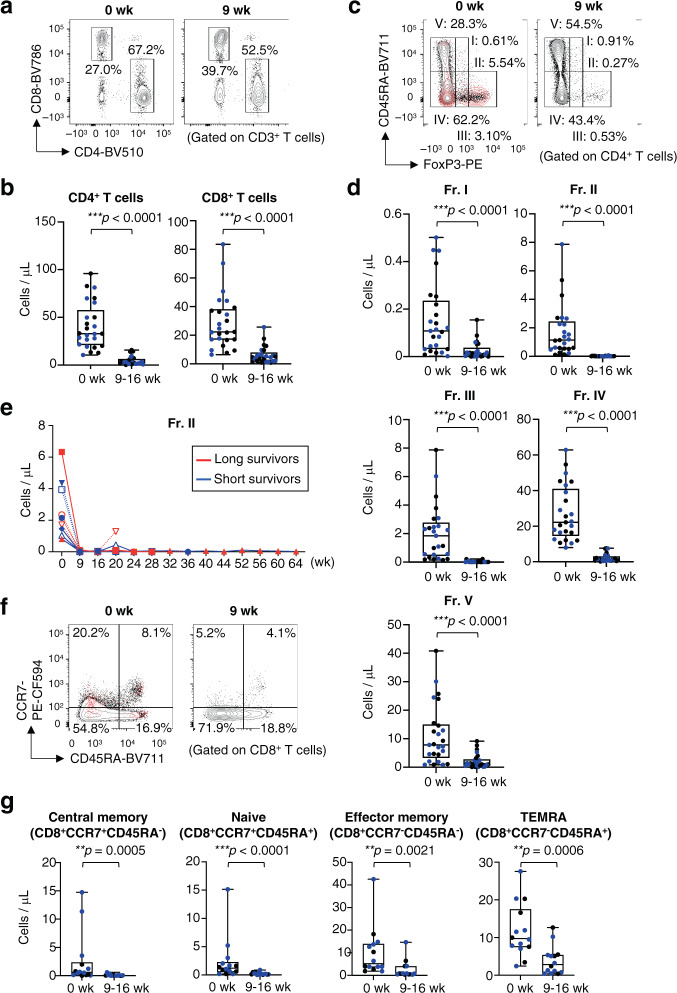
Mogamulizumab treatment reduces all T cell subsets in the peripheral blood. **a**, **b** Representative flow cytometry staining for CD4 and CD8 in CD3^+^ T cells (**a**) and summaries for the absolute number of CD4^+^ T cells (***p < 0.0001) and CD8^+^ T cells (***p < 0.0001) at pre- and post-mogamulizumab treatment (**b**) are shown. PBMCs (*n* = 25) obtained from pre- and post-mogamulizumab treatment. Pre-treatment samples were collected within two weeks before the initial mogamulizumab administration, and post-treatment samples were collected at 9-16 weeks after mogamulizumab administration and were subjected to flow cytometry. **c** Representative flow cytometry staining for CD45RA and FoxP3 in CD4^+^ T cells. Red dots, CCR4^+^ cells; black dots, CD4^+^ T cells. **d** Summaries for the absolute number of each CD4^+^ T cell fraction (Fr. I, ***p < 0.0001; Fr. II, ***p < 0.0001; Fr. III, ***p < 0.0001; Fr. IV, ***p < 0.0001 and Fr. V, ***p < 0.0001) as depicted in (**c**) at pre- and post-mogamulizumab treatment are shown (*n* = 25). **e**. Longitudinal changes in the absolute number of Treg cells (Fr. II) in CD4^+^ T cells at pre- and post-mogamulizumab treatment are shown. Red lines, long survivors; blue lines, short survivors. **f, g**. Representative flow cytometry staining for CD45RA and CCR7 in CD8^+^ T cells (**f**) and summaries for the absolute number of each CD8^+^ T cell fraction (Central memory, **p = 0.0005; Naive, ***p < 0.0001; Effector memory, *p = 0.0021 and TEMRA, **p = 0.0006) as depicted in (**f**) at pre- and post-mogamulizumab treatment (**g**) are shown (*n* = 14). Red dots, CCR4^+^ cells; black dots, CCR4^-^ cells. In (**b**, **d** and **g**), black dots, patients who received 1.0 mg/kg mogamulizumab; blue dots, patients who received 0.1 mg/kg mogamulizumab and two-sided Mann–Whitney test was used. In (**b**, **d** and **g**), the center line indicates the median, and the box limits indicate the 1st and 3rd quartiles. The whiskers go down to the smallest value and up to the largest. week: wk, naive CD8^+^ T cells: Naive, central memory CD8^+^ T cells: Central memory, effector-memory CD8^+^ T cells: Effector memory, TEMRA CD8^+^ T cells: TEMRA, *p < 0.01, **p < 0.001, ***p < 0.0001. Source data are provided as a Source Data file.

**Fig. 3:**
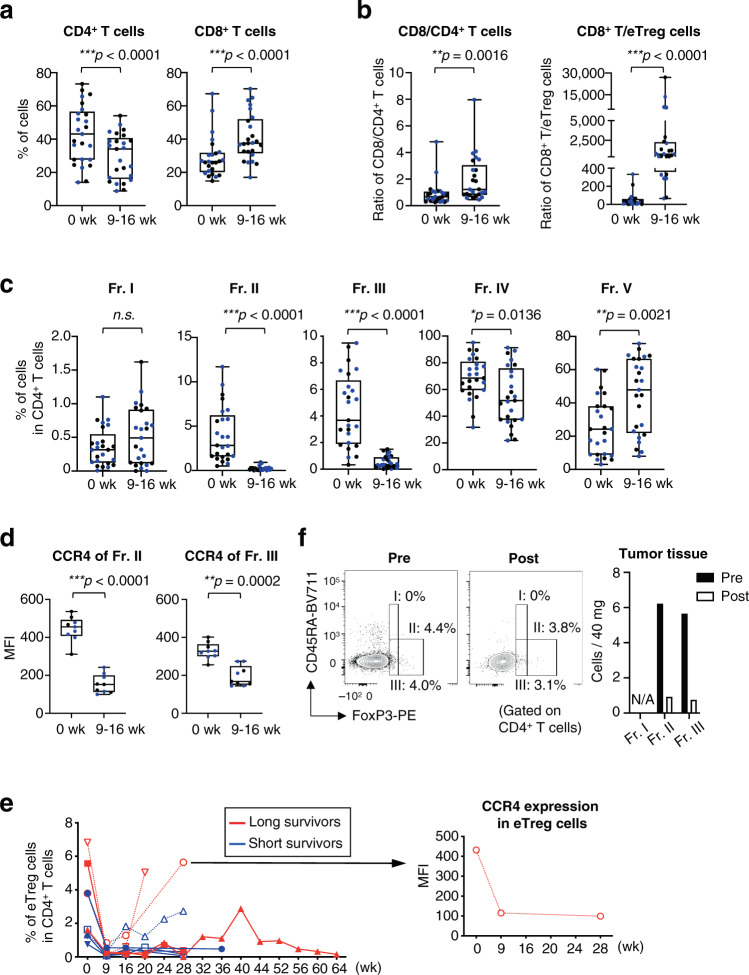
Mogamulizumab efficiently depletes eTreg cells in both peripheral blood and tumor tissues. **a** Summaries for the frequencies of CD4^+^ T cells (***p < 0.0001) and CD8^+^ T cells (***p < 0.0001) at pre- and post-mogamulizumab treatment are shown. PBMCs (*n* = 25) obtained from pre- and post-mogamulizumab treatment as in Fig. 2 and were subjected to flow cytometry. **b** The ratio of CD8^+^ T cells to CD4^+^ T cells (**p = 0.0016) or eTreg cells (***p < 0.0001) with the absolute number of the cells at pre- and post-mogamulizumab treatment is shown. **c** Summaries for the frequencies of each CD4^+^ T cell fraction (Fr. II, ***p < 0.0001; Fr. III, ***p < 0.0001; Fr. IV, *p = 0.0136 and Fr. V, **p = 0.0021) as depicted in Fig. 2c at pre- and post-mogamulizumab treatment are shown. PBMC samples (*n* = 25) as in (**a**) were subjected to flow cytometry in (**b** and **c**). **d** Changes in CCR4 expression levels of CCR4^+^CD4^+^ T cells (Fr. II, ***p < 0.0001 and Fr. III, **p = 0.0002) according to the mean fluorescence intensity (MFI) at pre- and post-mogamulizumab treatment are shown (*n* = 9). **e** Longitudinal changes in the frequencies of Treg cells (Fr. II) in CD4^+^ T cells at pre- and post-mogamulizumab treatment are shown (*n* = 9). Red lines, long survivors; blue lines, short survivors. **f** Fresh tumor samples (10 mg / approximately 4 ×4 x 4 mm^3^) obtained from a gastric cancer patient by endoscopic biopsy at pre- and post-mogamulizumab administration were subjected to flow cytometry. Flow cytometry staining for CD45RA and FoxP3 in CD4^+^ T cells (left) and changes of the absolute number of each FoxP3^+^ T cell fraction (right) are shown. In (**a–d**), black dots, patients who received 1.0 mg/kg mogamulizumab; blue dots, patients who received 0.1 mg/kg mogamulizumab and two-sided Mann–Whitney test was used. In **a–d**, the center line indicates the median, and the box limits indicate the 1st and 3rd quartiles. The whiskers go down to the smallest value and up to the largest. week: wk. *p < 0.05, **p < 0.005, ***p < 0.0001. Source data are provided as a Source Data file.

To confirm these findings, we examined additional PBMCs with flow cytometry using CD8^+^ T-cell and CD4^+^ T-cell subset gates (Supplementary Fig. 3)^21^. The absolute number of CD4^+^ T cells and CD8^+^ T cells was decreased after mogamulizumab administration in all patients tested (Fig. 2a, b). As the absolute number of all T-cell subsets was reduced after mogamulizumab treatment (Fig. 2a–g), the changes of immunological status such as CD8^+^ T-cell rich or Treg-cell rich were addressed via interrogating the frequency and the ratio of each T cell subset in CD4^+^ T cells and CD8^+^ T cells in detail. We then examined the frequency and the ratio of CD8^+^ T cells to CD4^+^ T cells or eTreg cells after mogamulizumab treatment, and found that the percentage of CD8^+^ T cells and the ratio of CD8^+^ T cells to CD4^+^ T cells or eTreg cells were increased (Fig. 3a, b), suggesting the dominance of CD8^+^ T cells after mogamulizumab treatment. To further explore the populations influenced by mogamulizumab administration, we divided CD4^+^ T cells into five fractions as previously reported^9,13,20^. CCR4-expressing Fr. II eTreg cells and Fr. III non-Treg cells were significantly decreased, while some CD4^+^ T cell populations particularly naive CD45RA^+^CD4^+^ T cells was significantly increased after mogamulizumab administration (Fig. 3c), reflecting the expression of CCR4; CCR4 expression was mainly detected in Fr. II eTreg cells and also in Fr. III non-Treg cells with low FoxP3 expression (Fig. 3d)^13,21^. In addition, the frequency of Fr. I naive Treg cell population was increased though the increase did not meet the pre-defined difference in statistical significance (Fig. 3c). These results confirmed the reduction of cluster 8 and 10 in CyTOF analyses. From some patients with a long follow-up, we could sequentially collect PBMC samples. Most patients, except some patients with loss of CCR4 expression in the Treg cells, showed a long-lasting reduction in eTreg cells during mogamulizumab administration in the absolute number and the frequency (Fig. 2e and Fig. 3e). Furthermore, we were able to collect tumor tissues pre- and post- mogamulizumab treatment from a gastric cancer patient. eTreg cells were markedly decreased after mogamulizumab administration in the absolute number and the frequency (Fig. 3f). Therefore, mogamulizumab, regardless of the dosage tested (0.1 and 1.0 mg/kg), can be considered to effectively deplete eTreg cells in the peripheral blood and probably in tumors, although the loss of CCR4 expression may hamper the effect.

### Central memory CD8^+^ T cells are reduced by mogamulizumab treatment

**Fig. 4:**
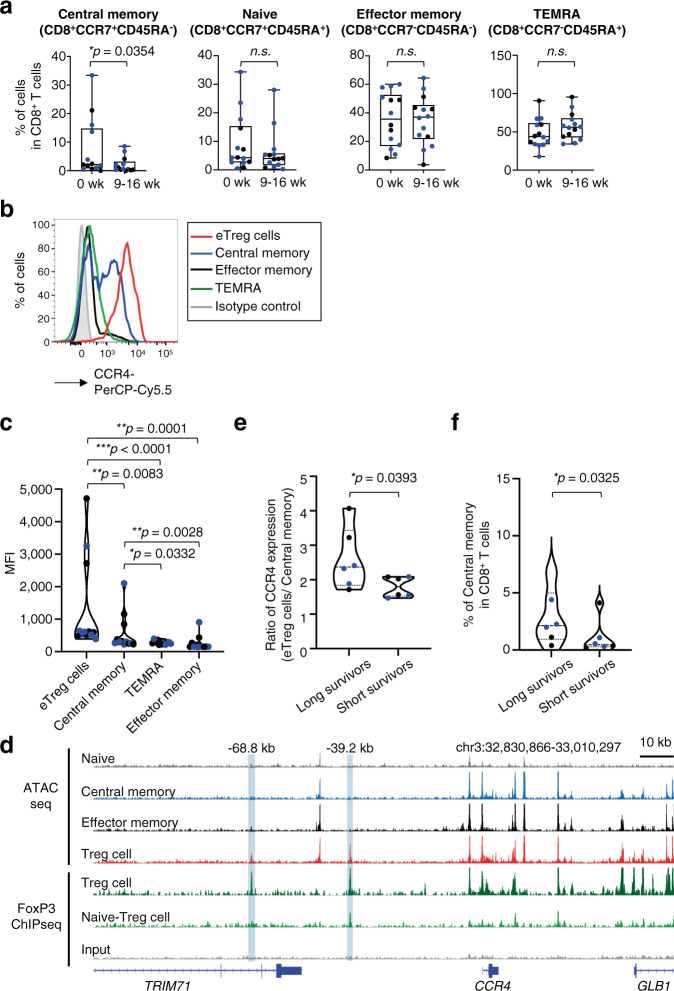
Central memory CD8^+^ T cells with CCR4 expression are decreased after mogamulizumab treatment. **a** Summaries for the frequencies of each CD8^+^ T cell fraction (central memory CD8^+^ T cells, *p = 0.0354) as depicted in Fig. 2f at pre- and post-mogamulizumab treatment are shown. PBMC samples (*n* = 14), as in Fig. 2, were subjected to flow cytometry. **b**, **c** Representative flow cytometry staining for CCR4 in each CD8^+^ T cell fraction in comparison with that in eTreg cells (**b**) and summary for the CCR4 expression levels (MFI) in central memory CD8^+^ T cells (to eTreg cells, **p = 0.0083), effector-memory CD8^+^ T cells (to eTreg cells, **p = 0.0001 and to central memory CD8^+^ T cells, **p = 0.0028), TEMRA CD8^+^ T cells (to eTreg cells, ***p < 0.0001 and to central memory CD8^+^ T cells, *p = 0.0332) and eTreg cells (**c**) are shown (*n* = 12). Red line, eTreg cells; blue line, central memory CD8^+^ T cells; black line, effector-memory CD8^+^ T cells; green line, TEMRA CD8^+^ T cells; filled gray area, control staining. **d** Sequencing tracks of ATAC-seq regarding four T cell subsets (naive CD8^+^ T cells, central memory CD8^+^ T cells, effector-memory CD8^+^ T cells and Treg cells) and FoxP3 ChIP-seq about Treg cells around the CCR4 gene locus. The normalized ATAC-seq and ChIP-seq read coverage were used to visualize the tracks. ATAC seq about each T cell subset (gray: naive CD8^+^ T cells, blue: central memory CD8^+^ T cells, black: effector-memory CD8^+^ T cells, red: Treg cells) and FoxP3 ChIP-seq about Treg cells (green: Treg cells, light green: naive Treg cells, grey: IP control) are shown. **e** Ratio of CCR4 expression levels (mean fluorescence intensity: MFI) in central memory CD8^+^ T cells to eTreg cells (*p = 0.0393) in long survivors (≥1 year, *n* = 6) and short survivors (< 1 year, n = 6) is shown. **f** The frequency of central memory CD8^+^ T cells in CD8^+^ T cells after mogamulizumab treatment (*p = 0.0325) in long survivors and short survivors is shown (*n* = 6). In (**a**, **c**, **e** and **f**), black dots, patients who received 1.0 mg/kg mogamulizumab; blue dots, patients who received 0.1 mg/kg mogamulizumab. In (**a** and **c**), two-sided, (**e** and **f**), one-sided Mann–Whitney test was used. In **a**, the center line indicates the median, and the box limits indicate the 1st and 3rd quartiles. The whiskers go down to the smallest value and up to the largest. week: wk, naive CD8^+^ T cells: Naive, central memory CD8^+^ T cells: Central memory, effector-memory CD8^+^ T cells: Effector memory, TEMRA CD8^+^ T cells: TEMRA. *p < 0.05, **p < 0.005, ***p < 0.0001. Source data are provided as a Source Data file.

Based on the clinical failure of mogamulizumab treatment and the possible reduction in the subpopulation(s) of CD8^+^ T cells (particularly cluster 12) according to the CyTOF analyses (Fig. 1), we extensively explored the changes in detailed CD8^+^ T cell populations. As the absolute number of all CD8^+^ T-cell subsets was reduced after mogamulizumab treatment (Fig. 2f, g), the changes in the frequency of CD8^+^ T-cell subsets were examined. The frequency of central memory CD8^+^ T cells was significantly reduced after mogamulizumab administration (pre: mean 7.5%, post: mean 2.0%), although that of CD8^+^ T cells as a whole was significantly increased (Fig. 2a, f, 3a, and 4a). In addition, increased frequencies of effector-memory CD8^+^ T cells and terminally-differentiated effector-memory (TEMRA) CD8^+^ T cells was found though the increase did not meet the pre-defined difference in statistical significance (Fig. 2f and 4a)^26^. In accordance with this, central memory CD8^+^ T cells (mean value: 565), but not effector-memory CD8^+^ T cells (mean value: 255) or TEMRA CD8^+^ T cells (mean value: 274) expressed CCR4, although the level of expression was significantly lower than that of eTreg cells (mean: 1299) (Fig. 4b, c). The different expression levels could partly be attributed to an increase in chromatin accessibility in eTreg cells compared to that in central memory CD8^+^ T cells, as detected by the ATAC-seq peaks; an open chromatin region that was only identified by Treg cells was compatible with the binding site of FoxP3 (Fig. 4d), which is the master transcription factor of Treg cells^8,9^. In addition, CD4^+^ T cells transduced with *FoxP3* gene induced high CCR4 expression in FoxP3^high^ CD4^+^ T-cell fraction (Supplementary Fig. 4a). In addition, CCR4 expression by MJ, a FoxP3-expressing adult T cell leukemia/lymphoma (ATLL) cell line, which highly expresses CCR4^27^, was reduced by RNA interference-mediated knockdown of FoxP3 gene (Supplementary Fig. 4b). These results suggest that FoxP3 may be involved in the enhancement of CCR4 expression in Treg cells.

We next explored the distinct impacts of mogamulizumab on central memory CD8^+^ T cells in relation to clinical responses, given the important role of central memory CD8^+^ T cells in antitumor immunity^24,25^. To address the potential for mogamulizumab binding, the ratio of CCR4 expression in eTreg cells to central memory CD8^+^ T cells was analyzed. As the Kaplan-Meyer curve for overall survival showed a tail-plateau shape after 300 days, we determined the cut point for long survivors and short survivors as 12 months. The ratio of CCR4 expression in Treg cells to central memory CD8^+^ T cells was significantly lower in short survivors (<1 year) than in long survivors (≥1 year) (Fig. 4e). By contrast, the frequency of eTreg cells was comparably reduced in most patients in spite of survival time after mogamulizumab treatment (Fig. 3e). Accordingly, the frequency of central memory CD8^+^ T cells was significantly higher in long survivors compared to that in short survivors (Fig. 4f). Thus, central memory CD8^+^ T cells that express CCR4 are concomitantly reduced along with eTreg cells by mogamulizumab treatment (0.1 and 1.0 mg/kg), particularly in short survivors.

### NK cells show an exhausted phenotype in long survivors

**Fig. 5:**
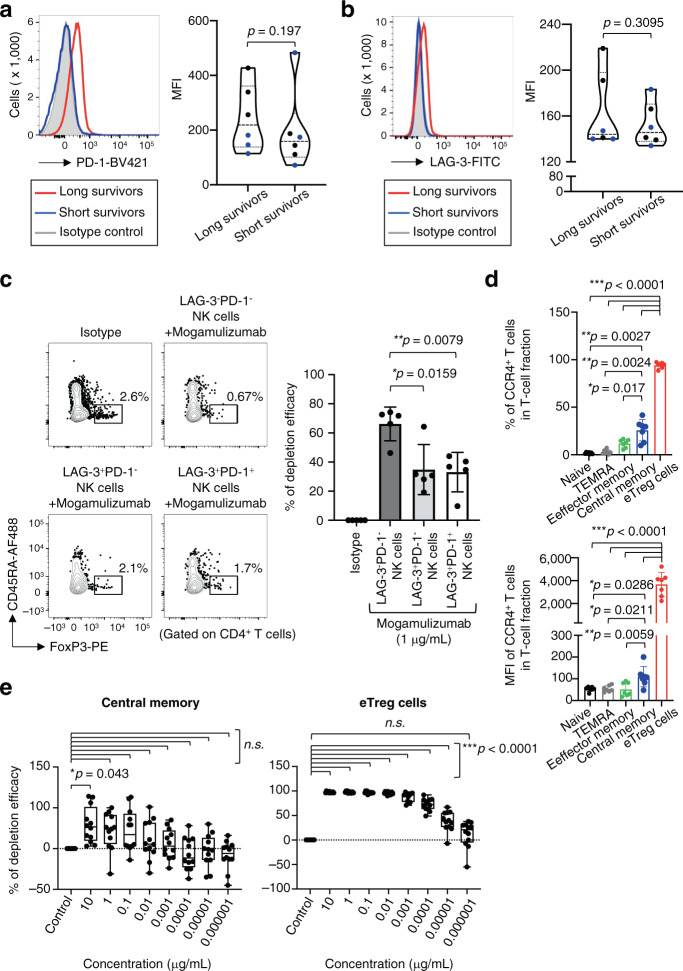
NK cells exhibit an exhausted phenotype in long survivors. **a**, **b** Representative flow cytometry staining (left) for PD-1 (**a**) and LAG-3 (**b**) in NK cells and summaries (right) for the frequencies of NK cells at pre-mogamulizumab treatment (PD-1, *p* = 0.197 and LAG-3, *p* = 0.3095) in long survivors (≥1 year) and short survivors (< 1 year) are shown. PBMC samples (long survivors: *n* = 6, short survivors: *n* = 6), as in Fig. 4, were subjected to flow cytometry. **c** The distinct ADCC activity by exhausted NK cells. LAG-3^-^PD-1^-^, LAG-3^+^PD-1^-^ and LAG-3^+^PD-1^+^ NK cells derived from PBMCs of healthy individuals (*n* = 5) were co-cultured with CD4^+^ T cells in the presence of mogamulizumab, and reduction of eTreg cells was examined. Representative flow cytometry data (left) and summary for depletion efficacy of eTreg cells (LAG-3^+^PD-1^-^ NK cells, *p = 0.0159 and LAG-3^+^PD-1^+^ NK cells, **p = 0.0079) are shown (right). **d** The frequencies of CCR4-expressing cells (top) and expression levels (mean fluorescence intensity: MFI) of CCR4 (bottom) in each CD8^+^ T cell fraction and eTreg cells in PBMCs from healthy individuals (*n* = 7). The differences between each CD8^+^ T cell fraction and eTreg cells were ***p < 0.0001 in the frequencies and the expression levels. The differences between central memory CD8^+^ T cells and Naive CD8^+^ T cells, and effector memory CD8^+^ T cells, and TEMRA CD8^+^ T cells were **p = 0.0027, *p = 0.017 and **p = 0.0024, respectively in the frequencies of CCR4-expressing cells; were *p = 0.0286, **p = 0.0059 and *p = 0.0211, respectively in the expression levels of CCR4. **e** Reduction of central memory CD8^+^ T cells (left) and eTreg cells (right) after mogamulizumab treatment. PBMC samples from healthy individuals (*n* = 12) were cultured with the indicated dose of mogamulizumab. Changes in each T cell fraction were examined (10 µg/mL, *p = 0.043 in central memory CD8^+^ T cells and 10–0.00001 µg/mL, ***p < 0.0001 in eTreg cells). In (**a** and **b**), black dots, patients who received 1.0 mg/kg mogamulizumab; blue dots patients who received 0.1 mg/kg mogamulizumab. Naive CD8^+^ T cells: Naive, central memory CD8^+^ T cells: Central memory, effector-memory CD8^+^ T cells: Effector memory, TEMRA CD8^+^ T cells: TEMRA. In (**a** and **b**), one-sided Mann–Whitney test was used. In (**c**), two-sided Mann–Whitney test was used. In (**d** and **e**), two-sided Dunnett test was used. In (**e**), the center line indicates the median, and the box limits indicate the 1st and 3rd quartiles. The whiskers go down to the smallest value and up to the largest. *p < 0.05, **p < 0.005, ***p < 0.0001. Source data are provided as a Source Data file.

Given the uncoupling of clinical efficacy and CCR4 expression in eTreg cells and central memory CD8^+^ T cells, we further explored the potential involvement of NK cell function in the clinical efficacy of mogamulizumab treatment because mogamulizumab possesses enhanced antibody-dependent cellular cytotoxicity (ADCC) activity via a defucosylated Fc region^28^. NK cells exhibited an exhausted phenotype, which was determined by the expression of PD-1 and LAG-3, in some patients particularly in long survivors, although the differences did not meet the pre-defined statistical significance (Fig. 5a, b), implying the presence of impaired ADCC activity in long survivors^29^. Patients, from whom the data regarding both CCR4 expression and NK cell exhaustion were available, were divided into long survivors and short survivors, and CCR4 expression and NK cell exhaustion were compared. The potential association between long survivors and lower expression of CCR4 in central memory CD8^+^ T cells/ higher levels of NK cell exhaustion was suggested (Supplementary Fig. 5). To further confirm the function of exhausted NK cells in ADCC activity, NK cells with or without exhausted phenotypes were cultured with CD4^+^ T cells, and the efficacy of eTreg cell reduction was examined. eTreg cells were reduced when cultured with non-exhausted NK cells (LAG-3^−^PD-1^−^NK cells), whereas the reduction was significantly attenuated in co-culture with exhausted NK cells (LAG-3^+^PD-1^−^ or LAG-3^+^PD-1^+^ NK cells) (Fig. 5c). Taken together, as ADCC activity in patients harboring exhausted NK cells is downregulated, eTreg cells with high CCR4 expression may be dominantly targeted by mogamulizumab treatment. By contrast, enhanced ADCC activity in patients with activated (non-exhausted) NK cells may kill both CCR4-expressing Treg cells and central memory CD8^+^ T cells by mogamulizumab treatment, irrespective of CCR4 expression level.

### Central memory CD8^+^ T cells and eTreg cells exhibit different sensitivity to mogamulizumab treatment

To gain a mechanistic insight into the in vivo data, PBMCs from healthy individuals were cultured with the titrated concentrations (10–0.000001 μg/ mL) of mogamulizumab reflecting the in vivo dosages based on the pharmacokinetics data from previous clinical trials^22,28,30^. Both eTreg cells and central memory CD8^+^ T cells were significantly reduced after adding mogamulizumab at 10 μg/ mL, which reflected the dosage of 1.0 mg/kg in vivo. However, less than 1 μg/ mL (less than 0.1 mg/kg in vivo) mogamulizumab selectively depleted eTreg cells compared with central memory CD8^+^ T cells, indicating that eTreg cells are preferentially targeted by mogamulizumab due to their higher CCR4 expression than that of central memory CD8^+^ T cells (Fig. 5d, e). To further examine the notion that central memory CD8^+^ T cells and eTreg cells were distinctly reduced at various mogamulizumab doses, we performed in vivo humanized mouse experiments in which mogamulizumab was administered to NSG mice transferred with human PBMCs, yet this in vivo animal model may require a careful interpretation, considering the short engraftment time. At 1.0 mg/kg dose of mogamulizumab, both eTreg cells and central memory CD8^+^ T cells were decreased, whereas a lower rate of reduction in central memory CD8^+^ T cells was observed at 0.1 and 0.01 mg/kg doses compared to eTreg cells (Supplementary Fig. 6). Altogether, the unexpected decrease in central memory CD8^+^ T cells with CCR4 expression could play a role in the low clinical efficacy of mogamulizumab in solid cancer patients, considering the relatively favorable clinical courses in patients who had central memory CD8^+^ T cells with lower CCR4 expression than eTreg cells and/or in whom NK cells exhibited an exhausted phenotype, which avoided the unexpected depletion of antitumor effector components.

## Discussion

Treg cell-targeted therapies have been investigated in the clinical setting with high expectations. Despite of the promising preclinical studies^10–13,31^, no Treg cell-targeted therapy has been successfully applied in the clinic. The poor clinical outcome was thought to be attributed to the low specificity of the reagents used for Treg cell deletion^13^. While this anti-CCR4 mAb (mogamulizumab) therapy was intended to target Treg cells, particularly eTreg cells, which have the highest expression of CCR4, most patients who were treated with mogamulizumab did not exhibit tumor regression, as observed in our previous phase 1a study^22^. Our comprehensive immune-monitoring assays revealed that the expression of CCR4 was not as selective as previously thought^21,22^, and central memory CD8^+^ T cells with CCR4 expression were concomitantly reduced along with Treg cells after mogamulizumab administration. Consequently, it is important to optimize the amount of mogamulizumab for achieving selective reduction of eTreg cells to avoid unexpected impairment of central memory CD8^+^ T cells^21,24,25,32^, leading to more effective immunotherapy. Given the importance of T cell clonal replacement rather than the reinvigoration of pre-existing activated T cells after ICB, as well as genetic transfection into non-activated (relatively naive) CD8^+^ T cells instead of effector cells in adoptive T cell therapies^24,25,33–35^, mogamulizumab may concurrently deplete harmful Treg cells and beneficial central memory CD8^+^ T cells that must be evoked and activated upon Treg cell depletion, cancelling antitumor clinical efficacy.

Mogamulizumab was approved for the treatment of CCR4-positive ATLL caused by human T-lymphotropic virus type 1 (HTLV-1) and peripheral T cell lymphoma (PTCL) in Japan in 2012^28,35^, and has also been tested for another HTLV-1-associated disease, HTLV-1-associated myelopathy-tropical spastic paraparesis (HAM-TSP)^30^. Additionally, in 2018, the FDA and EMA approved mogamulizumab as a treatment for patients with two subtypes of cutaneous T cell lymphoma (CTCL), namely, mycosis fungoides and Sézary syndrome, who have received at least one prior systemic therapy^36^. In dose-escalation studies for the treatment of ATLL and HAM-TSP, lower dosages (0.003–0.03 mg/kg) of mogamulizumab were examined and a significant reduction of Treg cells in the peripheral blood was observed, as was observed in this study^22,28,30^. In the treatment of CCR4-positive ATLL, PTCL and CTCL, mogamulizumab works as a “molecular-targeted reagent” that directly kills malignant cells via ADCC: The target cells (CCR4-positive cells) of mogamulizumab must be abundant in the host, compared to the present study in which mogamulizumab was used for the treatment of advanced or recurrent solid (CCR4-negative) tumors as a “cancer immunotherapy reagent” that kills eTreg cells. One can envision that excessive doses of mogamulizumab targeted central memory CD8^+^ T cells with CCR4 expression, although these cells showed much lower expression of CCR4 than eTreg cells due to differences in their epigenetic status; a low dosage of mogamulizumab, such as 0.01 mg/kg–0.001 mg/kg, at which Treg cell reduction in the peripheral blood was observed^30^, may be sufficient and optimal for Treg cell depletion to avoid the concomitant reduction of central memory CD8^+^ T cells. Indeed, our in vitro and in vivo experiments showed the relatively selective depletion of Treg cells at lower concentrations. In addition, patients who experienced clinical benefits exhibited a smaller reduction of central memory CD8^+^ T cells: in these patients, central memory CD8^+^ T cells harbored lower CCR4 expression and/or NK cells exhibited an exhausted phenotype, strongly suggesting the avoidance of mogamulizumab-mediated ADCC. Moreover, in a phase 1 dose-escalation study on ATLL patients, durable clinical responses, which are often observed in response to cancer immunotherapy were reportedly observed at lower dosages (0.01 mg/kg) than the dosages (0.1 and 1.0 mg/kg) applied in the clinic and in the present study^28^.

The maximum tolerated dose is the highest dose of a drug that does not cause unacceptable side effects; this is generally employed as the optimal dose for cytotoxic anticancer reagents. However, in cancer immunotherapy, the maximum tolerated dose may not always be the optimal dose. When mogamulizumab is used for Treg cell depletion as a “cancer immunotherapy reagent”, doses lower than the maximum tolerated dose could provide favorable clinical outcomes. However, as the patient samples tested in our study were limited, it is warranted to conduct a wide range of dose-escalation studies considering durable clinical responses and dose-de-escalation studies with comprehensive immunological analyses.

Another plausible explanation for the impairment of clinical responses to mogamulizumab in advanced or recurrent solid (CCR4-negative) tumor patients is that mogamulizumab did not sufficiently deplete eTreg cells in the TME. Recent studies that addressed comprehensive gene expression profiling in tumor-infiltrating Treg cells in colorectal, non-small-cell lung and breast cancers have revealed that CCR4 is not the optimal molecule for targeting tumor-infiltrating Treg cells; this is because CCR4 is predominantly expressed by eTreg cells in the peripheral blood rather compared to in the TME^37,38^. We have previously shown that CCR4 is highly expressed by tumor-infiltrating Treg cells in melanoma patients^21^. As CCR4 acts as a skin-homing chemokine receptor, tumor-infiltrating Treg cells in skin lesions of melanoma may possess high CCR4 expression^32^. In addition, NK cell activity is crucial for ADCC medicated by mogamulizumab, which harbors enhanced ADCC activity via its defucosylated Fc region and is often impaired in the TME. Nevertheless, we observed a marked reduction of eTreg cells in the TME in a gastric cancer patient, although studies with a large number of patients are warranted. Moreover, after long-term observation of eTreg cell reduction by mogamulizumab, eTreg cell rebound was observed in a few patients. Given that those rebounded eTreg cells possessed decreased expression of CCR4 and eTreg cells are composed of diverse populations^37,38^, this rebound may be explained by an increase of CCR4-negative eTreg cells. In other words, it should be emphasized that Treg cells are heterogeneous, when considering Treg cell-targeted therapy.

An original animal study showing the potential application of Treg cell-targeted cancer immunotherapy implicates the limited window for Treg cell-targeted therapy; Treg cell depletion induces tumor regression in some tumor lines, such as Meth A and RL-male 1, but not in others, such as AKSL2 and RL-female8^14^. Thus, we need to determine biomarker(s) that can identify the tumors in which Treg cells are essential for survival and growth by clarifying the immune suppressive network controlled by Treg cells, as has been done for ICB therapies^5,6^. Our previous study has illustrated the potential application of Treg cell-targeted therapy in high-risk patients for hyperprogressive diseases upon PD-1 blockade therapy^39^.

In conclusion, the unexpected decrease in central memory CD8^+^ T cells with CCR4 expression accompanied by Treg cell reduction due to the use of excessive doses of mogamulizumab was observed in solid cancer patients who received mogamulizumab monotherapy. This reduction in central memory CD8^+^ T cells was more striking in short survivors compared to long survivors. Therefore, we recommend that the optimal dose of mogamulizumab for cancer immunotherapy should be carefully determined. Efficient depletion of tumor-infiltrating eTreg cells observed in a gastric cancer patient encourages us to examine mogamulizumab as a Treg cell depletion reagent in further studies with large cohorts for clinical application.

## Methods

### Patients

Patients over 20 years old with advanced or recurrent CCR4-negative cancer were enrolled in this study from October 2013 to April 2015. CCR4 expression was determined by immunohistochemistry with anti-CCR4 mAb as described in Immunohistochemistry. Eligibility criteria included a good performance status (an Eastern Cooperative Oncology Group performance status of 0–2) and the following laboratory values: absolute neutrophil count ≥1,500/μL, hemoglobin ≥8.0 g/dL, platelet count ≥ 75,000/μL, total bilirubin ≤2.0 mg/dL, AST ≤ 2.5 × the upper limit of the normal range (UNL), ALT ≤ 2.5 × UNL, serum creatinine ≤1.5 mg/dL, and arterial blood oxygen saturation ≥ 93%. All patients underwent electrocardiography to confirm the absence of cardiac abnormalities requiring therapeutic intervention and that the left ventricular ejection fraction was at least 50%. Patients were excluded if they had an active infection, a history of organ transplantation, active concurrent cancer, any autoimmune diseases, central nervous system involvement, hepatitis B or C virus infection, HIV infection or previous ICB therapy.

### Study design

This multi-institutional, open-label, two-arm, phase Ib study is a part of an investigator-initiated phase Ia/Ib clinical trial of mogamulizumab administration in patients with CCR4-negative advanced or recurrent solid tumors (NCT01929486)^23^. No specific cancer type was selected since this is a Phase 1 study as the primary objectives were to characterize the safety and the effect of Treg cell depletion in peripheral blood. The secondary objectives were to assess the antitumor activity and to determine the recommended phase II dose. Twenty and nineteen patients were randomly enrolled in cohorts treated with dosages of 1.0 and 0.1 mg/kg mogamulizumab, respectively, and received the drug weekly for 8 weeks, which was followed by monthly intravenous infusion until disease progression was observed. These two dosages of mogamulizumab were determined as the maximum-tolerated dose and minimal dose in our previous phase Ia study^22^.

Toxicity was graded according to the National Cancer Institute Common Terminology Criteria for Adverse Events (version 4.0). Clinical responses were evaluated at 12 weeks after the first mogamulizumab administration or at the point of study discontinuation using computed tomography scans. The effects were determined according to the RECIST criteria (version 1.1). Progression-free survival (PFS) was defined from the day of the first mogamulizumab administration until the day of progressive disease (PD) detection. Peripheral blood samples were serially collected, and PBMCs were isolated by density gradient centrifugation with Ficoll-Paque (GE Healthcare, Little Chalfont, UK). Due to the ethical issues (because all patients enrolled in this study harbored advanced solid tumors and were often in anemic state), we were only able to obtain a sufficient amount of samples as the indicated number of patients in each figure. To collect TILs, fresh tumor tissues were minced and treated with a gentleMACS Dissociator (Miltenyi Biotec, Bergisch Gladbach, Germany) as previously described^39,40^, and the prepared cells were subjected to immune-monitoring assays.

The protocol was approved by the institutional review boards (Ethics Review Board of National Cancer Center, Ethics Review Board of Osaka University, Ethics Review Board of Nagoya University, Ethics Review Board of Aichi Medical University) at each participating site, and all patients provided written informed consent before enrollment in accordance with the Declaration of Helsinki.

### Immunohistochemistry

Biopsy samples were formalin-fixed, paraffin-embedded, and sectioned before they were placed onto slides for immunohistochemistry, which was conducted with anti-CCR4 mAb (KM2160; Kyowa Hakko Kirin) as previously reported^22^. CCR4 positivity was evaluated by the review committee with central evaluation.

### CyTOF analyses

CyTOF staining and analysis were performed as described^41^. The antibodies used in the CyTOF analyses are summarized in Supplementary Table 2. Cells were subjected to staining after washing with PBS supplemented with 2% fetal calf serum (FCS, Biosera, Orange, CA, USA) (washing solution). The cells were incubated in 5 μM of Cell-ID rhodium solution (Fluidigm, South San Francisco, CA) in PBS, washed using washing solution, and stained with a mixture of surface-staining antibodies (1:100 dilution). After washing, the cells were fixed and permeabilized using a Foxp3/Transcription Factor Staining Buffer Set (Thermo Fisher Scientific, Waltham, MA) according to the manufacturer’s instructions. The fixed and permeabilized cells were stained with the intracellular antibodies (1:50 dilution). After washing twice, the cells were allowed to rest overnight in 125 nM MaxPar Intercalator-Ir (Fluidigm) diluted in PBS solution with 2% paraformaldehyde at 4°C. The cells were then washed once with washing solution and twice with MaxPar water (Fluidigm) and distilled water with minimal heavy element contamination to reduce the background level. The cells were resuspended in MaxPar water supplemented with 10% EQ Four Element Calibration Beads (Fluidigm) and then were applied to the Helios instrument (Fluidigm), and data were acquired at a speed below 300 events/second.

### Preprocessing and analysis of CyTOF data

The data from the pre- and post-mogamulizumab administration samples were combined for each patient, and batch effects were removed using ResNet^42^. After removing the batch effects, the data were combined, and the UMAP projections were generated using the R package “umap”. A modified version of CYBERTRACK was used for clustering the CyTOF data^43^. The cluster sizes were determined by the Elbow method, and debris clusters were removed for further analyses.

### Flow cytometry analyses

Flow cytometry staining and analyses were performed as described^21,41,44^. The antibodies used in the flow cytometry analyses are summarized in Supplementary Table 3. Cells (PBMCs and TILs) were washed using washing solution and subjected to staining with surface antibodies (1:50 dilution). Intracellular staining of FoxP3 was performed with anti-FoxP3 mAb (1:50 dilution) and Foxp3/Transcription Factor Staining Buffer Set (Thermo Fisher Scientific) according to the manufacturer’s instructions. After washing, the cells were analyzed with an LSRFortessa instrument (BD Biosciences, San Jose, CA), FACSDiva software (ver.8.0.1, BD Biosciences), FlowJo software (ver.10, BD Biosciences) and Excel for Mac 16 (Microsoft). The dilution of the staining antibodies was performed according to the manufacturer’s instructions.

### ATAC-seq data processing

ATAC-seq data reported by Calderon et. al^45^ were employed. Law FASTQ files about naive CD8^+^ T cells, central memory CD8^+^ T cells, effector-memory CD8^+^ T cells (these CD8^+^ T cell subsets were not further stimulated in vitro) and Treg cells were downloaded from the Gene Expression Omnibus (accession no. GSE118189). Trim-Galore (https://www.bioinformatics.babraham.ac.uk/projects/trim_galore/) was utilized for trimming adaptor sequence and filtering low quality reads. The filtered FASTQ files were aligned to GRCh38 genome using Bowtie2 v2.4.1^46^. The reads mapped to chrM, mapping quality < 30 and with the flags “- F 1804” and “-f 2” were filtered out using samtools v1.9 (http://www.htslib.org), and the duplicated reads were also removed using Picard Tools v1.119 (https://broadinstitute.github.io/picard/). Then, the resultant BAM files for each cell type were merged. Chromatin accessibility signals were calculated from merged BAM files using deepTools2^47^. For normalizing signals, we used reads counts data mapped to ATAC peaks regions detected by MACS2^48^ under the parameters “-nonodel -nolamda -keep-dup all -call summits”. The MACS2 narrow peak files from each cell type were merged using bedtools2 (https://bedtools.readthedocs.io/en/latest/). The mapped reads in merged peaks region were counted using featureCounts^49^. The size factors were calculated using R “DESeq” package, and then ATAC signals were normalized. For visualizing ATAC signals, Integrative Genomics Viewer was used^50^.

### ChIP-seq data processing

FoxP3 ChIP-seq data reported by Birzele et. al. and Schmldl et al. were employed^51,52^. Law FASTQ files about Treg cells and naive Treg cells were downloaded from the NCBI SRA (SRP006674) and GEO (GSE43119), respectively. Trim-Galore was used for trimming adaptor sequence and filtering low quality reads. We then aligned filtered FASTQ files to GRCh38 genome using Bowtie2 v2.4.1^46^. The duplicated reads and reads having mapping quality < 4 were filtered out using Picard Tools v1.119 and samtools v1.9, respectively. The resultant BAM files for each cell type were merged. FoxP3 ChIP signals were calculated from merged BAM files using deepTools2 under the parameters “-bs = 10 –normalize Using CPM --extendReads 200”^47^. For visualizing FoxP3 ChIP-seq signals, Integrative Genomics Viewer was used^50^.

### T cell culture

PBMCs from healthy individuals were cultured with in a round-bottom 96-well plate with medium containing IL-2 (10 U/mL, Peprotech) and IL-7 (20 ng/mL, Peprotech). The indicated dose of mogamulizumab was added to some wells during the entire culture period. After 5 days, the cells were subjected to flow cytometry staining and analyses.

### ADCC activity by NK cells

CD56^+^ cells prepared from human PBMCs were cultured with mitomycin C-treated K562 cell line in the presence of anti-NKG2C antibody (10 µg/mL, clone: 134522, R&D systems), IL-2 (100 IU/mL, Peprotech) and IL-15 (10 ng/mL, Peprotech) for one week^53^. Then, LAG-3^−^PD-1^−^, LAG-3^+^PD-1^−^ and LAG-3^+^PD-1^+^ NK cells were sorted with a FACSymphony™ S6 Cell Sorter (BD Biosciences) and co-cultured with CD4^+^ T cells in the presence of mogamulizumab.

### Statistical analysis

The relationships between groups were compared using a *t*-test or the nonparametric Mann–Whitney *U* test. For multiple group comparisons, the Dunnett test was employed. PFS and OS were defined as the time from the initial mogamulizumab administration until the first observation of disease progression and death from any cause, respectively. PFS and OS were investigated with the Kaplan–Meier method and were compared among the groups using the log-rank test or Cox regression proportional hazards analysis. Statistical analysis was performed with GraphPad Prism8 and 9 (GraphPad Software, San Diego, CA) or R version 3.1.1 (R Foundation for Statistical Computing, Vienna, Austria). *P* values less than 0.05 were considered significant.

### Reporting summary

Further information on research design is available in the Nature Research Reporting Summary linked to this article.

## Supplementary information

Supplementary Information

Reporting Summary

## Data Availability

The ATAC-seq data regarding four T cell subsets and FoxP3 ChIP-seq data used in this study are available GRCh38 genome and in the NCBI database under accession code “GSE118189”, “GSE43119” and “SRP006674”. Raw data that support the findings of this study are available from the corresponding author (H.N.) upon reasonable request. Source data in this study are provided as a Source Data file. Source data are provided with this paper.

## Source data

Source Data

## Code availability

Associated Code can be found here: https://github.com/kodaim1115/Maeda_CCR4.
