# Supplementary material for: Depletion of central memory CD8+ T cells might impede the antitumor therapeutic effect of Mogamulizumab
Source: Nat Commun. 2021 Dec 14;12:7280. doi: 10.1038/s41467-021-27574-0 (PMC8671535; doi:10.1038/s41467-021-27574-0)
Supplement: Supplementary file 1 — Supplementary Information [file 41467_2021_27574_MOESM1_ESM.pdf]

Title: Depletion of Central Memory CD8$^+$ T Cells Might Impede the Antitumor Therapeutic Effect of Mogamulizumab

Authors: Yuka Maeda[1], Hisashi Wada[2*], Daisuke Sugiyama[3], Takuro Saito[4], Takuma Irie[1], Kota Itahashi[1], Kodai Minoura[5], Susumu Suzuki[6], Takashi Kojima[7], Kazuhiro Kakimi[8], Jun Nakajima[9], Takeru Funakoshi[10], Shinsuke Iida[11], Mikio Oka[12], Teppei Shimamura[5], Toshihiko Doi[7], Yuichiro Doki[4], Eiichi Nakayama[13], Ryuzo Ueda[6*] and Hiroyoshi Nishikawa[1,3*]

Supplementary Information

Supplementary Fig. 1: Clinical courses of patients with solid cancers treated with mogamulizumab.

Supplementary Fig. 2: The entire landscape of changes at pre- and post- mogamulizumab treatment.

Supplementary Fig. 3: The gating strategy for T cell assays.

Supplementary Fig. 4: Control of CCR4 expression by FoxP3 in CD4$^+$ T cells and ATLL cell line.

Supplementary Fig. 5: NK-cell exhaustion and CCR4 expression in T cell subsets are potentially associated with patient survival.

Supplementary Fig. 6: Central memory CD8$^+$ T cells and eTreg cells exhibit different sensitivity to mogamulizumab treatment.

Supplementary Table 1. Patient characteristics and clinical responses who were examined with CyTOF.

Supplementary Table 2. Antibodies and tetramers used in the CyTOF analyses.

Supplementary Table 3. Antibodies and tetramers used in the flow cytometry analyses.

Supplementary methods

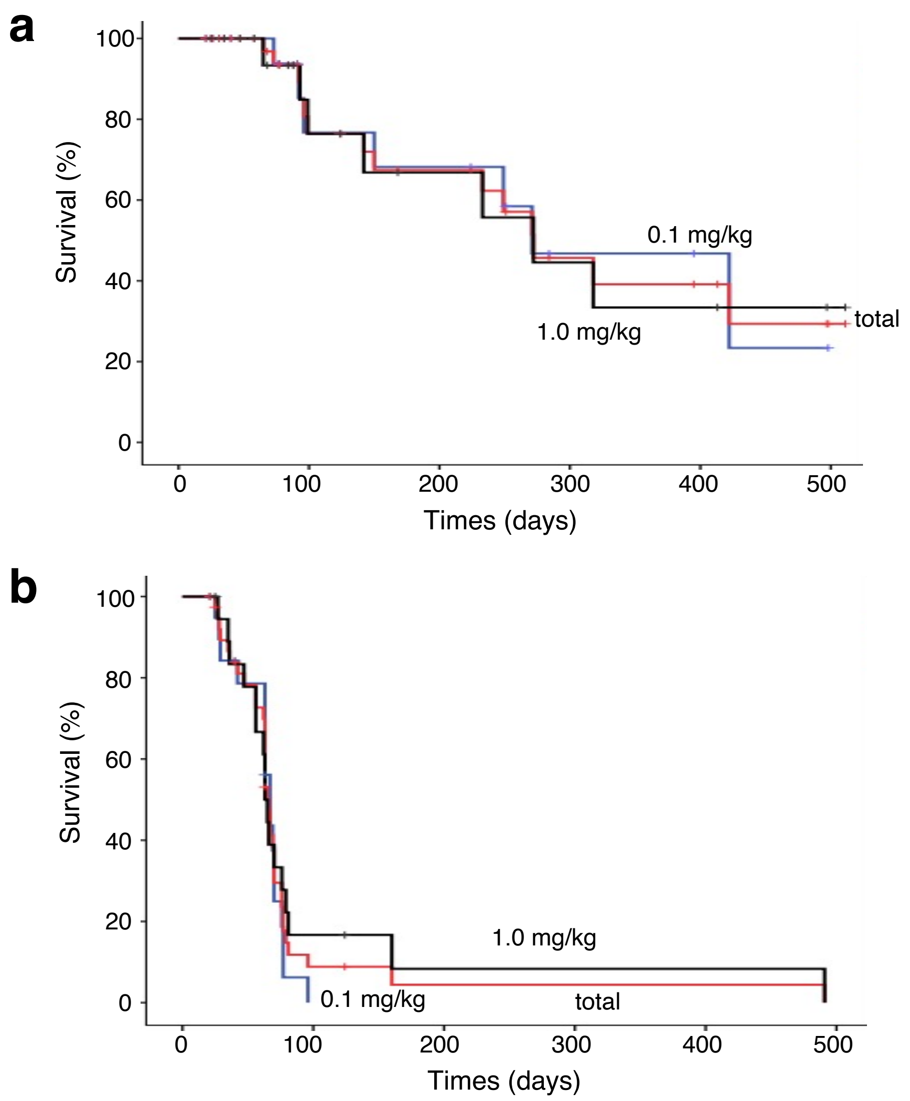

Supplementary Fig. 1: Clinical courses of patients with solid cancers treated with mogamulizumab.

a, b. Kaplan-Meier curves of OS (**a**) and PFS (**b**) for 39 CCR4-negative solid cancer patients (11 esophageal cancer, 12 lung cancer, 6 malignant melanoma, 5 gastric cancer and 5 ovarian cancer patients) who were treated with mogamulizumab (0.1 or 1.0 mg/kg). Green lines, total patients; red line, patients who received 1.0 mg/kg mogamulizumab; blue lines, patients who received 0.1 mg/kg mogamulizumab. Source data are provided as a Source Data file.

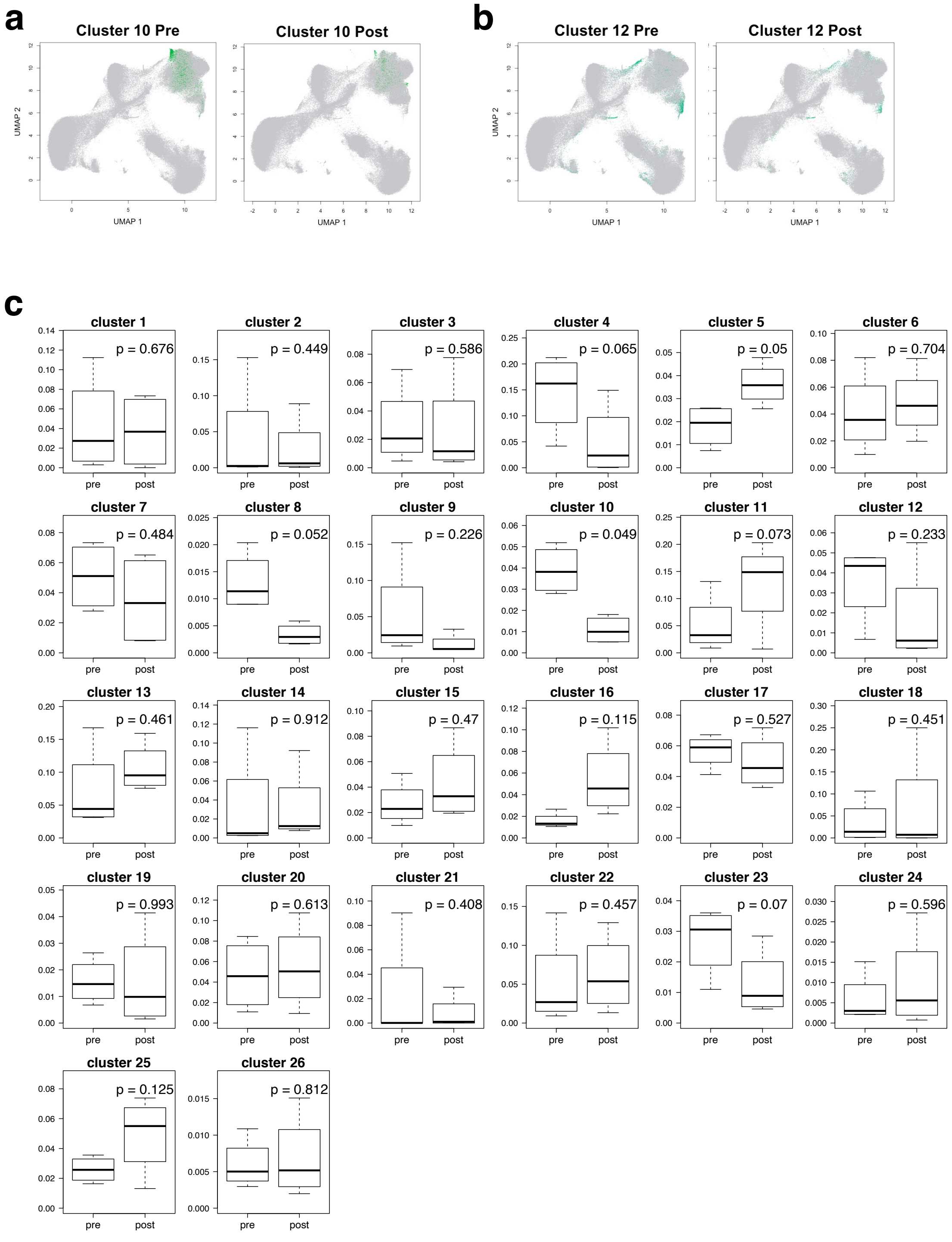
a
Cluster 10 Pre
Cluster 10 Post
b
Cluster 12 Pre
Cluster 12 Post
UMAP 1
UMAP 2
c
cluster 1 p = 0.676
cluster 2 p = 0.449
cluster 3 p = 0.586
cluster 4 p = 0.065
cluster 5 p = 0.05
cluster 6 p = 0.704
cluster 7 p = 0.484
cluster 8 p = 0.052
cluster 9 p = 0.226
cluster 10 p = 0.049
cluster 11 p = 0.073
cluster 12 p = 0.233
cluster 13 p = 0.461
cluster 14 p = 0.912
cluster 15 p = 0.47
cluster 16 p = 0.115
cluster 17 p = 0.527
cluster 18 p = 0.451
cluster 19 p = 0.993
cluster 20 p = 0.613
cluster 21 p = 0.408
cluster 22 p = 0.457
cluster 23 p = 0.07
cluster 24 p = 0.596
cluster 25 p = 0.125
cluster 26 p = 0.812
pre
post

Supplementary Fig. 2: The entire landscape of changes at pre- and post-mogamulizumab treatment.

a, b. UMAP projection with green color indicating cells assigned to cluster 10 (Treg cells) (**a**) and cells assigned to cluster 12, which comprised the $CD8^+$ T cell population in the mixed population of $CD4^+$ and $CD8^+$ T cells (**b**). **c**. Boxplot of pre- and post-treatment proportions of all clusters (clusters 1, $p = 0.676$; 2, $p = 0.449$; 3, $p = 0.586$; 4, $p = 0.065$; 5, $p = 0.05$; 6, $p = 0.704$; 7, $p = 0.484$; 8, $p = 0.052$; 9, $p = 0.052$; 10, $*p = 0.049$; 11, $p = 0.071$; 12, $p = 0.233$; 13, $p = 0.461$; 14, $p = 0.912$; 15, $p = 0.47$; 16, $p = 0.115$; 17, $p = 0.527$; 18, $p = 0.451$; 19, $p = 0.993$; 20, $p = 0.613$; 21, $p = 0.408$; 22, $p = 0.457$; 23, $p = 0.07$; 24, $p = 0.596$; 25, $p = 0.125$; 26, $p = 0.812$) detected by CYBERTRACK2.0 in CyTOF analysis using PBMCs (n=4). The center line indicates the median, and the box limits indicate the 1st and 3rd quartiles. Whiskers extend to the 1.5x interquartile range. Two-sided paired Student's t-test was used for the comparison of changes at pre- and post-mogamulizumab treatment. Source data are provided as a Source Data file.

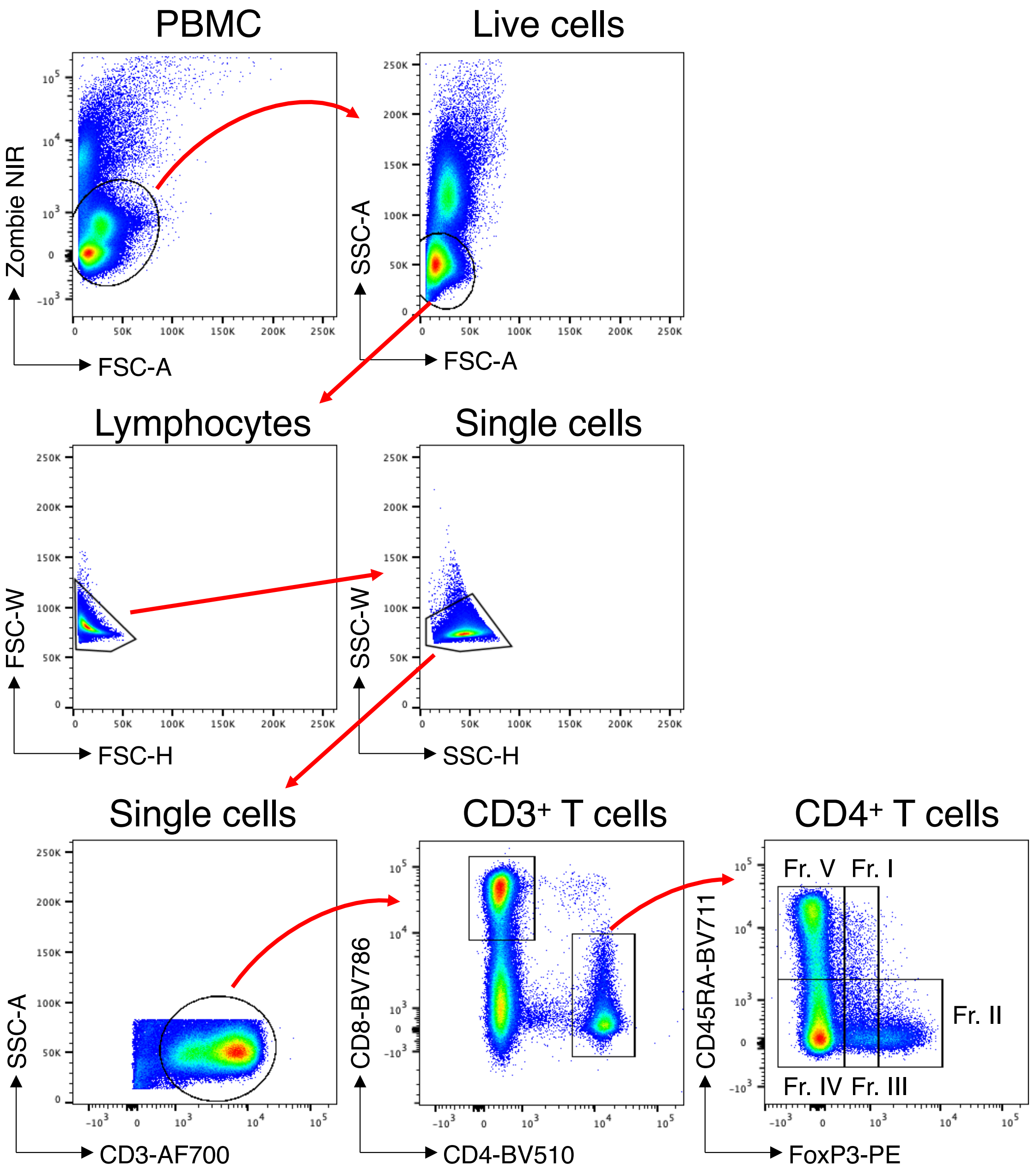
PBMC
Live cells
Lymphocytes
Single cells
Single cells
CD3+ T cells
CD4+ T cells
Zombie NIR
FSC-A
SSC-A
FSC-A
FSC-W
FSC-H
SSC-W
SSC-H
SSC-A
CD3-AF700
CD8-BV786
CD4-BV510
CD45RA-BV711
FoxP3-PE
Fr. V
Fr. I
Fr. II
Fr. IV
Fr. III

Supplementary Fig. 3: The gating strategy for T cell assays.

The live cells were identified as gating live/dead marker negative cells, and live lymphocytes were defined by gating FSC-A vs. SSC-A. Singlets were identified by exclusion of doublets using an FSC-H vs. FSC-W plot and SSC-H vs. SSC-W plot. Then, gates were set on $CD3^{+}$cells (T cells) and then $CD4^{+}CD8^{-}$ T cells and $CD4^{-}CD8^{+}$ T cells. $CD4^{+}$ T cells were further separated based on the expression of CD45RA and FoxP3 to identify each $CD4^{+}$T cell subpopulation.

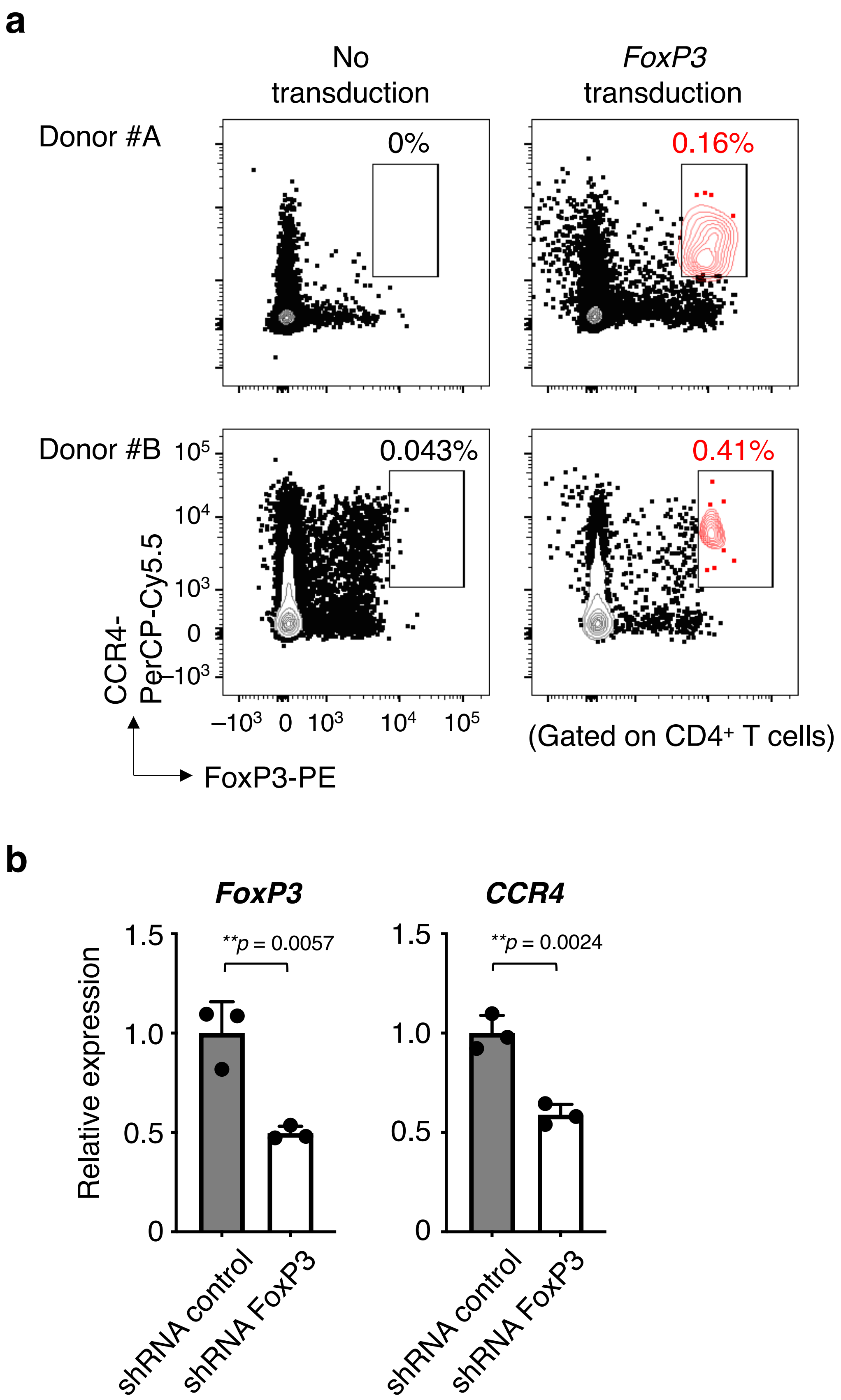
a
No transduction
FoxP3 transduction
Donor #A
0%
0.16%
Donor #B
0.043%
0.41%
CCR4-PerCP-Cy5.5
FoxP3-PE
(Gated on CD4+ T cells)
b
FoxP3
CCR4
Relative expression
**p = 0.0057
**p = 0.0024
shRNA control
shRNA FoxP3

Supplementary Fig. 4: Control of CCR4 expression by FoxP3 in $CD4^+$ T cells and ATLL cell line.

a. Unstimulated $CD4^+$ T cells from PBMCs of healthy individuals (n = 2) were transduced with *FoxP3* gene and were cultured with IL-2 (30 IU/mL) for three days. CCR4 expression was examined with flow cytometry. Red dots, $FoxP3^{high}CCR4^+$ cells; black dots, $CD4^+$ T cells. **b.** Knockdown of intracellular *FoxP3* in MJ, a FoxP3-expressing ATLL cell line, mediated by shRNAs was performed. Gene expression of *CCR4* (**p = 0.0057) and *FoxP3* (**p = 0.0024) in MJ cell lines was examined with real time-PCR. Error bars represent the standard deviation of triplicates. **b,** two-sided unpaired Student's t-test was used. The data represent the mean ± SD. **$p < 0.001$. Source data are provided as a Source Data file.

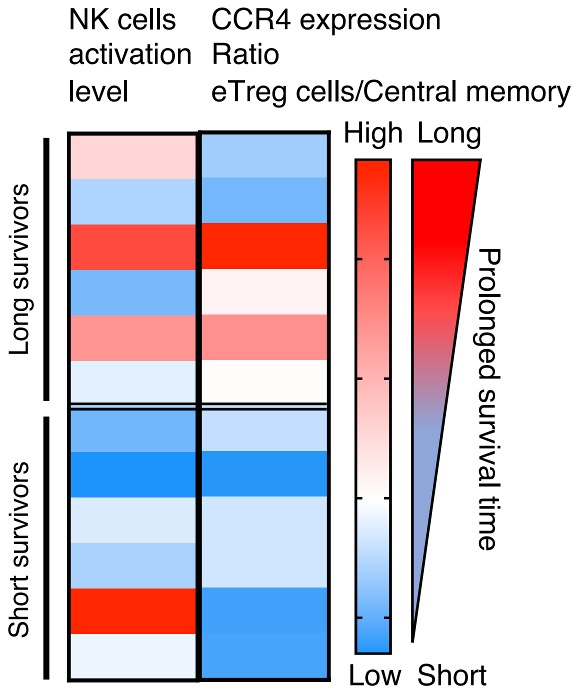

Supplementary Fig. 5: NK-cell exhaustion and CCR4 expression in T cell subsets are potentially associated with patient survival.

Heatmap of the frequency of exhausted NK cells and the ratios of CCR4 expression levels (mean fluorescence intensity: MFI) in eTreg cells to central memory $CD8^+$ T cells (Central memory). Source data are provided as a Source Data file.

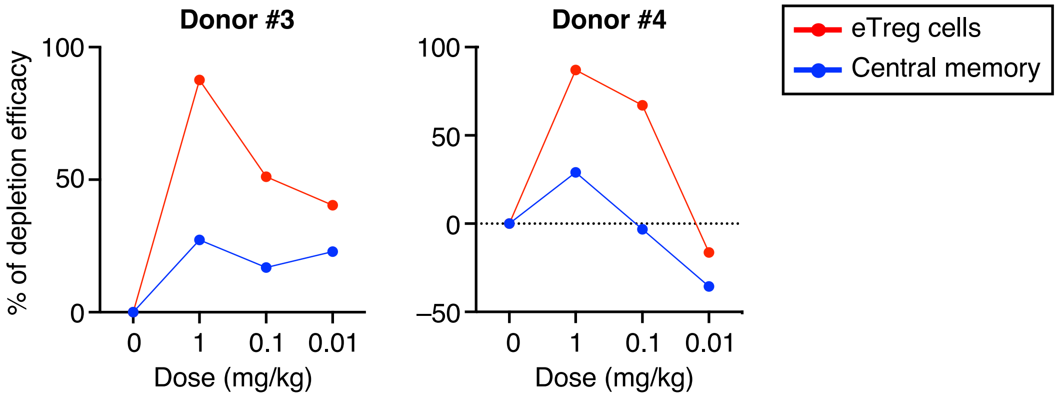

Supplementary Fig. 6: Central memory CD8$^+$ T cells and eTreg cells exhibit different sensitivity to mogamulizumab treatment.

NSG mice were administered with the indicated dose of mogamulizumab at one day after transferring PBMCs (1x10^7 cells / mouse) and spleen cells were collected from the mouse at three days after transferring PBMCs to examine with flow cytometry. The reduction of central memory CD8$^+$ T cells (Central memory) and eTreg cells was examined. Source data are provided as a Source Data file.

Supplementary Table 1. Patient characteristics and clinical responses who were examined with CyTOF.

	OUH-04	OUH-05	OUH-10	OUH-16
sex	Female	Male	Male	Female
age (year-old)	65	64	70	73
BMI (kg/m^2)	25.7	17.6	19.7	25.8
cancer organ	Ovary	Esophagus	Stomach	Ovary
clinical response	PD	PR	PD	PD
PFS (days) (RECIST)	66	491	70	76
OS (days)	413	511	271	84

Supplementary Table 2. Antibodies and tetramers used in the CyTOF analyses.

**Antibodies used for CyTOF**			
**Molecule**	**Clone**	**Company**	**Conjugation**
EOMES	WD1928	Fluidigm	141Pr
CD40	5C3	Fluidigm	142Nd
CD45RA	HI100	Fluidigm	143Nd
CD8a	RPA-T8	Fluidigm	146Nd
CD11c	Bu15	Fluidigm	147Sm
CD14	RMO52	Fluidigm	148Nd
pLck [T505]	4/LCK-Y505	Fluidigm	149Sm
CD134 (OX40)	ACT35	Fluidigm	150Nd
CD107a (LAMP1)	H4A3	Fluidigm	151Eu
CD194 (CCR4)	205410	Fluidigm	153Eu
CD3	UCHT1	Fluidigm	154Sm
CD279 (PD-1)	EH12.2H7	Fluidigm	155Gd
CD86	IT2.2	Fluidigm	156Gd
pStat3 [Y705]	4	Fluidigm	158Gd
FoxP3	236A/E7	Fluidigm	159Tb
Tbet	4B10	Fluidigm	160Gd
CD152 (CTLA-4)	14D3	Fluidigm	161Dy
CD80 (B7-1)	2D10.4	Fluidigm	162Dy
CD183 (CXCR3)	G025H7	Fluidigm	163Dy
CD185 (CXCR5)	51505	Fluidigm	164Dy
CD223 (LAG3)	874501	Fluidigm	165Ho
pNF-kB p65 [S529]	K10-895.12.50	Fluidigm	166Er
CD197 (CCR7)	G043H7	Fluidigm	167Er
CD154 (CD40L)	24-31	Fluidigm	168Er
CD25 (IL-2R)	2A3	Fluidigm	169Tm
CCR8	L263G8	Fluidigm	170Yb
CD20	2H7	Fluidigm	171Yb
pS6 [S235/S236]	N7-548	Fluidigm	172Yb
CD274 (PD-L1)	29E.2A3	Fluidigm	175Lu
CD4	RPA-T4	Fluidigm	176Yb

Supplementary Table 3. Antibodies and tetramers used in the flow cytometry analyses.

**Antibodies used for flow cytometry**			
**Molecule**	**Clone**	**Company**	**Conjugation**
Human CD3	UCHT1	BD Biosciences	AF-700
Human CD4	SK3	BD Biosciences	BV510
Human CD8	RPA-T8	BD Biosciences	BV786
Human CCR4	1G1	BD Biosciences	PerCP-Cy5.5
Human CD45RA	HI100	BioLegend	BV711<br>AF-488
Human PD-1	MIH4	BD Biosciences	BV421
Human FoxP3	236A/E7	eBioscience	PE
Human CTLA-4	BNI3	BD Biosciences	APC
Human LAG3	17B4	Enzo	FITC
Human CCR7	150503	BD Biosciences	PE-CF594
Zombie NIR		BioLegend	NIR

Supplementary methods

Lentivirus transduction to primary human CD4$^+$ T cells

Human *FoxP3* gene were inserted into pLVSIN-EF1α-Pur vector (Takara Bio, Shiga, Japan). In the lentiviral vector, *FoxP3*-pLVSIN-EF1α-Pur, psPAX2 (Addgene plasmid # 12260) and pMD2.G (Addgene plasmid # 12259) were combined at a ratio of 2:1:1, respectively, in Opti-MEM (Thermo Fisher Scientific, Waltham, MA). The DNA mixture was incubated with PEI-MAX (Polysciences, Inc, Warrington, PA) at a ratio of 1:3 for 15 minutes at room temperature. The complex was added to the Lenti-X 293T cell line. The culture supernatant was collected after 24 and 48 hour incubation and was passed through a 0.45 μm pore PVDF Millex-HV filter (Millipore, Billerica, MA). Concentrated lentivirus through centrifugation at 6000g overnight was added to the culture of primary human CD4$^+$ T cells, which were purified from PBMCs of healthy donors with a CD4$^+$ T Cell Isolation Kit (Miltenyi Biotec, Bergisch Gladbach, Germany). The transduced CD4$^+$ T cells were cultured with IL-2 (30 IU/mL) for 3 days, and were examined with flow cytometry.

Lentivirus transduction to ATLL cell line

FoxP3 knockdown in MJ, a FoxP3-expressing ATLL cell line, was performed with lentiviral transduction of FoxP3-shRNA vectors purchased from Vectorbuilder (VB900097-4153eup). Transduced cells were selected with puromycin at a concentration of 5 μg/ml for further analyses. MJ cells expressing a scrambled control shRNA were used for control (VB010000-0009mxc).

Real time-PCR

RNA was extracted from MJ cells transfected with control-shRNA or FoxP3-shRNA using a RNeasy Mini Kit (QIAGEN). cDNA was generated with a SuperScript IV VILO (Thermo Fisher Scientific), and RT-PCR was performed with PowerUp SYBR Green Master Mix (Thermo Fisher Scientific) using a QuantStudio 7 Flex Real-Time PCR System (Thermo Fisher Scientific). The gene expression was normalized with GAPDH as an endogenous control. The following PCR primers were used: GAPDH forward: GCACCGTCAAGGCTGAGAAC; GAPDH reverse: ATGGTGGTGAAGACGCCAGT; CCR4 forward: TGCTCTGCCAATACTGTGGG; ATGATCCATGGTGGACTGCG.

In vivo humanized mouse model

NSG (NOD.Cg-*Prkdc*^{scid} *Il2rg*^{tm1Wjl}/SzJ; 6-week-old females) mice were purchased from Charles River Laboratories (Yokohama, Japan). The mice were 6-10 week old female and housed in cages under specific pathogen free conditions, provided with sterilized standard food, given sterilized drinking water, and on a 12:12 light/dark cycle with lights on at 9:00 am. Temperature was kept at 22°C (20-26°C) and humidity at 45% (40-60%). Transferred with human PBMCs (1x10^7 cells / mouse) through tail vein. These mice were administered with the indicated dose of mogamulizumab at one day after transferring PBMCs. Spleens were collected from the mouse at three days after transferring PBMCs, and single-cell suspensions were prepared. The cells were analyzed with an LSRFortessa instrument (BD Biosciences) and FlowJo software (BD Biosciences). All mice were maintained in a specific pathogen-free facility at Nagoya University Graduate School of Medicine. Animal care and all experiments were conducted according to the guidelines of the Animal Committee of Nagoya University after approval by the Ethics Review Committee for Animal Experimentation of Nagoya University.
