# Supplementary material for: Depletion of central memory CD8+ T cells might impede the antitumor therapeutic effect of Mogamulizumab
Source: Nat Commun. 2021 Dec 14;12:7280. doi: 10.1038/s41467-021-27574-0 (PMC8671535; doi:10.1038/s41467-021-27574-0)
Supplement: Supplementary file 2 — Reporting Summary [file 41467_2021_27574_MOESM2_ESM.pdf]

nature portfolio

Corresponding author(s): Hiroyoshi Nishikawa, MD., PhD

Last updated by author(s): Nov 14, 2021

Reporting Summary

Nature Portfolio wishes to improve the reproducibility of the work that we publish. This form provides structure for consistency and transparency in reporting. For further information on Nature Portfolio policies, see our Editorial Policies and the Editorial Policy Checklist.

Statistics

For all statistical analyses, confirm that the following items are present in the figure legend, table legend, main text, or Methods section.

n/a	Confirmed	
☐	☒	The exact sample size (*n*) for each experimental group/condition, given as a discrete number and unit of measurement
☐	☒	A statement on whether measurements were taken from distinct samples or whether the same sample was measured repeatedly
☐	☒	The statistical test(s) used AND whether they are one- or two-sided<br>*Only common tests should be described solely by name; describe more complex techniques in the Methods section.*
☐	☒	A description of all covariates tested
☐	☒	A description of any assumptions or corrections, such as tests of normality and adjustment for multiple comparisons
☐	☒	A full description of the statistical parameters including central tendency (e.g. means) or other basic estimates (e.g. regression coefficient) AND variation (e.g. standard deviation) or associated estimates of uncertainty (e.g. confidence intervals)
☐	☒	For null hypothesis testing, the test statistic (e.g. *F*, *t*, *r*) with confidence intervals, effect sizes, degrees of freedom and *P* value noted<br>*Give P values as exact values whenever suitable.*
☒	☐	For Bayesian analysis, information on the choice of priors and Markov chain Monte Carlo settings
☒	☐	For hierarchical and complex designs, identification of the appropriate level for tests and full reporting of outcomes
☒	☐	Estimates of effect sizes (e.g. Cohen's *d*, Pearson's *r*), indicating how they were calculated

Our web collection on statistics for biologists contains articles on many of the points above.

Software and code

Policy information about availability of computer code

Data collection	FACSDiva software (ver.8.0.1), https://github.com/kodaim1115/Maeda_CCR4
Data analysis	Microsoft Excel for Mac 16, GrapfPad Prism 8 and 9, Flowjo (ver.10), CYBERTRACK2.0., R version 3.1.1, ResNet v0.1, UMAP v0.2, Trim-Galore v0.5, Bowtie2 v2.4.1, deepTools2, Integrative Genomics Viewer, Picard Tools v1.119, samtools v1.9, MACS2, bedtools2, featureCounts v1.6, DESeq v1.32, CPM --extendReads 200

For manuscripts utilizing custom algorithms or software that are central to the research but not yet described in published literature, software must be made available to editors and reviewers. We strongly encourage code deposition in a community repository (e.g. GitHub). See the Nature Portfolio guidelines for submitting code & software for further information.

Data

Policy information about availability of data

All manuscripts must include a data availability statement. This statement should provide the following information, where applicable:

- Accession codes, unique identifiers, or web links for publicly available datasets
- A description of any restrictions on data availability
- For clinical datasets or third party data, please ensure that the statement adheres to our policy

The ATAC-seq data about four T cell subsets and FoxP3 ChIP-seq data used in this study are available GRCh38 genome and in the NCBI database under accession code "GSE118189", "GSE43119" and "SRP006674".
Raw data that support the findings of this study are available from the corresponding author (H.N.) upon reasonable request. Source data in this study are provided as a Source Data file.

Field-specific reporting

Please select the one below that is the best fit for your research. If you are not sure, read the appropriate sections before making your selection.

- [x] Life sciences
- [ ] Behavioural & social sciences
- [ ] Ecological, evolutionary & environmental sciences

For a reference copy of the document with all sections, see nature.com/documents/nr-reporting-summary-flat.pdf

Life sciences study design

All studies must disclose on these points even when the disclosure is negative.

Sample size	The number of patients who enrolled in this Phase 1b trial was 39 and the sample size tested was the same.
Data exclusions	No data exclusions were needed.
Replication	All experiments were conducted at least 2 times with similar results.
Randomization	No randomization was performed in this study, because this study was a phase 1b study to characterize the safety and the effect of regulatory Tcell depletion in peripheral blood with mogamulizumab (anti-CCR4 mAb).
Blinding	No blinded experiments were conducted in this study, because this study was a phase 1b study to characterize the safety and the effect of regulatory Tcell depletion in peripheral blood with mogamulizumab (anti-CCR4 mAb).

Reporting for specific materials, systems and methods

We require information from authors about some types of materials, experimental systems and methods used in many studies. Here, indicate whether each material, system or method listed is relevant to your study. If you are not sure if a list item applies to your research, read the appropriate section before selecting a response.

Materials & experimental systems

n/a	Involved in the study	
[ ]	[x]	Antibodies
[ ]	[x]	Eukaryotic cell lines
[x]	[ ]	Palaeontology and archaeology
[ ]	[x]	Animals and other organisms
[ ]	[x]	Human research participants
[ ]	[x]	Clinical data
[x]	[ ]	Dual use research of concern

Methods

n/a	Involved in the study	
[x]	[ ]	ChIP-seq
[ ]	[x]	Flow cytometry
[x]	[ ]	MRI-based neuroimaging

Antibodies

Antibodies used

CyTOF:
141Pr- EOMES, Fluidigm, WD1928, Custom made
142Nd- CD40, Fluidigm, 5C3, 3142010B
143Nd- CD45RA, Fluidigm, HI100, 3143006B
146Nd- CD8a, Fluidigm, RPA-T8, 3146001B
147Sm- CD11c, Fluidigm, Bu15, 3147008B
148Nd- CD14, Fluidigm, RMO52, 3148010B
149Sm- pLck [T505], Fluidigm, 4/LCK-Y505, Custom made
150Nd- CD134 (OX40), Fluidigm, ACT35, 3150023B
151Eu- CD107a (LAMP1), Fluidigm, H4A3, 3151002B
153Eu- CD194 (CCR4), Fluidigm, 205410, 3153013A
154Sm- CD3, Fluidigm, UCHT1, 3154003B
155Gd- CD279 (PD-1), Fluidigm, EH12.2H7, 3155009B
156Gd- CD86, Fluidigm, IT2.2, 3156008B
158Gd- pStat3 [Y705], Fluidigm, 4, 3158005A
159Tb- FoxP3, Fluidigm, 236A/E7, Custom made
160Gd- Tbet, Fluidigm, 4B10, 3160010B
161Dy- CD152 (CTLA-4), Fluidigm, 14D3, 3161004B
162Dy- CD80 (B7-1), Fluidigm, 2D10.4, 3162010B
163Dy- CD183 (CXCR3), Fluidigm, G025H7, 3163004B
164Dy- CD185 (CXCR5), Fluidigm, 51505, 3164016B

165Ho- CD223 (LAG3), Fluidigm, 874501, 3165028B
166Er- pNF-kB p65 [S529], Fluidigm, K10-895.12.50, 3166006A
3
nature research | reporting summary April 2020
167Er- CD197 (CCR7), Fluidigm, G043H7, 3167009A
168Er- CD154 (CD40L), Fluidigm, 24-31, 3168006B
169Tm- CD25 (IL-2R), Fluidigm, 2A3, 3169003B
170Yb- CCR8, Fluidigm, L263G8, Custom made
171Yb- CD20, Fluidigm, 2H7, 3171012B
172Yb- pS6 [S235/S236], Fluidigm, N7-548, 3172008A
175Lu- CD274 (PD-L1), Fluidigm, 29E.2A3, 3175017B
176Yb- CD4, Fluidigm, RPA-T4, 3176010B
Flow cytometry:
AF-700- Human CD3, BD Biosciences, UCHT1, 561027
BV510- Human CD4, BD Biosciences, SK3, 562970
BV786- Human CD8, BD Biosciences, RPA-T8, 563823
PerCP-Cy5.5- Human CCR4, BD Biosciences, 1G1, 560726
BV711 and AF488- Human CD45RA, BioLegend, HI100, 304137
BV421- Human PD-1, BD Biosciences, MIH4, 564323
PE- Human FoxP3, eBioscience, 236A/E7, 72-5774-40
APC- Human CTLA-4, BD Biosciences, BNI3, 557301
FITC- Human LAG3, Enzo, 17B4, ALX-804-806F-C100
PE-CF594- Human CCR7, BD Biosciences, 150503, 562381
NIR- Zombie NIR, BioLegend, 423105
ADCC activity for NK cell:
Unconjugated Human NKG2C, R&D systems, 134522, MAB1381-100
In vivo and vitro experiment for T cell depletion:
Mogamulizumab, Kyowakirin, KW-0761
Ultra-LEAF Purified Human IgG1 Isotype control, BioLegend, QA16A12, 403501

Validation

These antibodies that are commercially available were validated by the manufacturers and used according to the manufacturer's instructions.
Concerning about reproducibility and validation,
BioLegend (https://www.biolegend.com/), BD Bioscience (https://www.bdbiosciences.com/en-us), eBioscience (https://www.thermofisher.com/jp/en/home/life-science/antibodies/ebioscience.html?gclid=EAIaIQobChMI15CKnNT58wIVQqqWCh14SAbmEAAYASAAEgJ2lPD_BwE&ef_id=EAIaIQobChMI15CKnNT58wIVQqqWCh14SAbmEAAYASAAEgJ2lPD_BwE:G:s&s_kwcid=AL!3652!3!376163228125!e!!g!!ebioscience&cid=bid_pca_aup_r01_co_cp1359_pjt0000_bid00000_0se_gaw_bt_pur_con), Fluidigm (https://www.fluidigm.com/), Enzo (https://www.enzolifesciences.com/browse/products/by-product-type/antibodies/) and R&D systems (https://www.rndsystems.com/?gclid=EAIaIQobChMIx7fQ79T58wIVAcuWCh2jzglbEAAYASAAEgLWLvD_BwE).

Eukaryotic cell lines

Policy information about cell lines

Cell line source(s)	K562 and MJ cell lines were obtained from ATCC and Lenti-X 293T cell line was obtained from TaKaRa bio.
Authentication	K562, MJ and Lenti-X 293T cells were not authenticated by genetic profiling.
Mycoplasma contamination	All cell lines were regulatory tested and negative for mycoplasma contamination.
Commonly misidentified lines (See ICLAC register)	No commonly misidentified cell lines were used in the study.

Animals and other organisms

Policy information about studies involving animals; ARRIVE guidelines recommended for reporting animal research

Laboratory animals	NSG mice (NOD.Cg-Prkdcscid Il2rgtm1Wjl/SzJ (Charles River Laboratories, Yokohama, Japan) were used for the in vivo studies. The mice were 6-10 week old female and housed in cages under specific pathogen free conditions, provided with sterilized standard food, given sterilized drinking water, and on a 12:12 light/dark cycle with lights on at 9:00 am. Temperature was kept at 22°C (20-26°C) and humidity at 45% (40-60%).
Wild animals	No wild animals were used in the study.
Field-collected samples	No field-collected samples were used in the study.
Ethics oversight	All mouse experiments were approved by the Animals Committee for Animal Experimentation of the Nagoya University Graduate School of Medicine ,Japan.

Note that full information on the approval of the study protocol must also be provided in the manuscript.

Human research participants

Policy information about studies involving human research participants

Population characteristics	Japanese patients (male, n=25; female, n=14; year-old, 45-85) with esophageal cancer (n=11), non-small cell lung cancer (n=12), malignant melanoma (n=6), gastric cancer (n=5) or ovarian cancer (n=5) were enrolled in this study. Uncharacterized PBMCs from healthy donors were purchased from C.T.L..
Recruitment	Solid tumor patients over 20 years old with advanced or recurrent CCR4-negative cancer were enrolled in this study from October 2013 to April 2016. CCR4 expression was determined by immunohistochemistry with anti-CCR4 mAb (KM2160; Kyowa Hakko Kirin). Healthy donors who consented to participate in the study were randomly selected, and PBMCs derived from these donors were used.
Ethics oversight	The protocol was approved by the institutional review boards (Ethics Review Board of National Cancer Center Hospital East, Ethics Review Board of Osaka University, Ethics Review Board of Nagoya University, Ethics Review Board of Aichi Medical University) at each participating site, and all patients provided written informed consent before enrolment in accordance with the Declaration of Helsinki.

Note that full information on the approval of the study protocol must also be provided in the manuscript.

Clinical data

Policy information about clinical studies

All manuscripts should comply with the ICMJEguidelines for publication of clinical research and a completedCONSORT checklist must be included with all submissions.

Clinical trial registration	NCT01929486
Study protocol	This multi-institutional, open-label, two-arm, phase Ib is a part of an investigator-initiated phase Ia/Ib clinical trial of mogamulizumab administration in patients with CCR4-negative advanced or recurrent solid tumors. (https://clinicaltrials.gov/ct2/show/NCT01929486)
Data collection	Clinical data were collected from medical records. Patients were recruited from August 28, 2013 to February 17, 2016, and data were collected by Aichi Medical University.
Outcomes	The effects were determined according to the RECIST criteria (ver. 1.1) and were published in Saito T, et al. Phase Ib study of a humanized anti-CCR4 antibody, KW-0761, in advanced solid tumors. Nagoya J Med Sci. in press.

Flow Cytometry

Plots

Confirm that:

- [x] The axis labels state the marker and fluorochrome used (e.g. CD4-FITC).
- [x] The axis scales are clearly visible. Include numbers along axes only for bottom left plot of group (a 'group' is an analysis of identical markers).
- [x] All plots are contour plots with outliers or pseudocolor plots.
- [x] A numerical value for number of cells or percentage (with statistics) is provided.

Methodology

Sample preparation	Peripheral blood samples were serially collected, and PBMCs were isolated by density gradient centrifugation with Ficoll-Paque (GE Healthcare, Little Chalfont, UK). To collect TILs, fresh tumor tissues were minced and treated with a gentleMACS Dissociator (Miltenyi Biotec, Bergisch Gladbach, Germany).
Instrument	LSRFortessa, Symphony S6 cell sorter (BD Biosciences, San Jose, CA), CyTOF (Fluidigm, South San Francisco, CA)
Software	FACSDiva software was used for data collection, and FlowJo v.10 was used for analysis.
Cell population abundance	The purity >90% after cell sorting was confirmed by flow cytometry.
Gating strategy	First we gated based on physical parameters (FSC-A vs SSC-A), then excluded doublets (FSC-H vs FSC-W and SSC-H vs SSC-W). On the singlets, we selected for live cells using Zombie NIR (BioLegend). CD8+ T cells as CD3+CD8+, CD4+ T cells as CD3+CD4 +, effector Treg cells as CD45RA-FoxP3highCD4+CD3+. In addition, the detailed method of gating is shown in Supplementary Fig. 3

- [x] Tick this box to confirm that a figure exemplifying the gating strategy is provided in the Supplementary Information.
